# Bisecting GlcNAc enhances CD8^+^ T cell-mediated killing of breast cancer by suppressing PD-L1 expression and its binding to PD-1

**DOI:** 10.1186/s40164-025-00693-w

**Published:** 2025-08-04

**Authors:** Xueting Ren, Jinpeng Wu, Jing Li, Zhen Zhai, Xiang Li, Feng Guan, Meng Wang, Xiaobin Ma, Zengqi Tan, Huafeng Kang, Shuai Lin

**Affiliations:** 1 The Comprehensive Breast Care Center, The Second Affiliated Hospital of Xi’an Jiaotong University, Xi’an, Shaanxi China; 2 Key Laboratory of Resource Biology and Biotechnology in Western China, Ministry of Education, College of Life Sciences, Northwest University, Xi’an, Shaanxi China; 3 Institute of Hematology, Provincial Key Laboratory of Biotechnology, School of Medicine, Northwest University, Xi’an, Shaanxi China

**Keywords:** Breast cancer, Bisecting GlcNAc, CD8^+^ T cells, PD-L1, Immunotherapy

## Abstract

**Background:**

The abundance of PD-L1 on the surface of tumor cells is a critical factor in sensitizing these cells to T cell-mediated immune killing. While abnormal glycosylation of PD-L1 is known to influence its expression and function, the precise regulatory mechanisms remain unclear.

**Methods:**

This study utilized bioinformatics analysis to explore the role of MGAT3, a key gene involved in the formation of the bisecting GlcNAc structure, in breast cancer (BC). Experimental approaches were employed to increase bisecting GlcNAc levels in BC cells, followed by assessments of PD-L1 expression, CD8^+^ T cell-mediated cytotoxicity, extracellular vesicle (EV)-associated PD-L1, and PD-L1/PD-1 interaction. Additionally, forskolin, a bisecting GlcNAc agonist, was combined with anti-PD-L1 antibody to evaluate its antitumor effects in vivo.

**Results:**

MGAT3 was found to be expressed at low levels in BC tissues but positively correlated with CD8^+^ T cell infiltration. Elevating bisecting GlcNAc levels in BC cells significantly enhanced the cytotoxic efficacy of CD8^+^ T cells. High bisecting GlcNAc modification promoted PD-L1 degradation via the lysosomal pathway, reducing PD-L1 expression and its binding to PD-1. Furthermore, increased bisecting GlcNAc levels reduced PD-L1 in tumor cell-derived EVs, impairing the EVs’ ability to block CD8^+^ T cells and indirectly enhancing T cell cytotoxicity. The combined use of forskolin and anti-PD-L1 antibody significantly increased CD8^+^ T cell abundance and activity, achieving a more effective antitumor response in vivo.

**Conclusions:**

These findings demonstrate that enhancing bisecting GlcNAc modification in BC cells promotes PD-L1 degradation and inhibits its binding to PD-1, thereby boosting CD8^+^ T cell-mediated cytotoxicity, providing a promising strategy for immune modulation in BC therapy.

**Supplementary Information:**

The online version contains supplementary material available at 10.1186/s40164-025-00693-w.

## Introduction

Breast cancer (BC) is now the most common malignant tumor worldwide and the leading cause of cancer-related deaths in women [1, 2]. In recent years, the treatment of BC has advanced significantly, moving from traditional methods such as surgery, chemotherapy, and radiotherapy, to targeted drugs, and has now entered a new era of immunotherapy [3]. Unlike traditional treatments, immunotherapy harnesses cytokines, chemokines and immune cells to remodel the tumor microenvironment (TME), thereby improving anti-tumor efficacy and preventing relapse [4]. In particular, the success of immune checkpoint inhibitors (ICIs), represented by PD-1/PD-L1 monoclonal antibodies (mAbs), in the field of BC treatment marks a new stage in BC immunotherapy [5]. However, the majority of patients fail to benefit from these therapies due to primary or acquired resistance resulting from tumor immune escape [6]. Therefore, in-depth exploration of the immune escape mechanism and the development of new strategies to overcome immune escape are crucial for improving immunotherapy response rates in BC patients [7].

Extracellular vesicles (EVs), derived from multivesicular bodies or from the plasma membrane which carry proteins, lipids, mRNAs and non-coding RNAs including microRNAs, with diameters ranging from 30 nm to 10 μm, and assist in intercellular communication [8, 9]. EVs are involved in numerous physiological processes, especially in immune regulation. EVs carrying tumor peptides presented by MHC class I and II complexes can directly stimulate CD4^+^ and CD8^+^ T cells, or indirectly stimulate T cells by interacting with antigen-presenting cells, playing immunostimulatory or immunosuppressive roles [10–15]. Notably, tumor cells can actively produce a large number of EVs enriched with pro-cancer cellular contents, such as immunosuppressive proteins represented by PD-L1. PD-L1 in EVs can recapitulate the action of cell surface PD-L1 and promote tumor progression by similarly inhibiting T-cell activation to allow cancer cells to evade anti-tumor immunity [16]. It is evidenced that EVs have the potential to act as therapeutic agents for regulating the immune system, and therapies based on EVs are being developed and clinical trials are under way for the treatment of inflammatory diseases, autoimmune diseases and cancer.

As a highly glycosylated immune protein, the glycosylation not only affects PD-L1 stability but also its binding affinity with PD-1, thereby contributing crucially to immune evasion [17, 18]. Glycosylation, as the most common and complex type of post-translational modification (PTM), plays a specific role in recognizing and mediating multiple physiological and pathological processes [19]. Abnormal glycosylation, as hall marker of malignancy, is known to be crucial in regulating key oncogenic processes, including malignant transformation, invasion, metastasis and angiogenesis [20]. Abnormal glycosylation can also regulate tumor immunity by altering the immune system’s recognition of tumor cells, and induce immune evasion by affecting the binding of glycosylated receptors and ligands on the cell surface [21]. For example, the interaction between sialylated glycans and sialic acid-binding immunoglobulin-like lectin can induce tumor-promoting phenotypes in tumor-associated macrophages, inhibit the activation of natural killer cells and neutrophils, reduce dendritic cell maturation and antigen presentation, and inhibit T cell responses [22]. In addition, most immune checkpoint molecules are glycoproteins whose structure and biological functions are largely affected by glycosylation status. For instance, N-glycosylation can reduce the proteasomal degradation of PD-L1 and maintain its interaction with PD-1, thereby promoting T cell immune escape [17], whereas O-GlcNAcylation can facilitate tumor immune evasion by suppressing the lysosomal degradation of PD-L1 [23]. Our previous studies found that overexpression of MGAT3 increased the level of bisecting GlcNAc and inhibited the proliferation and migration of BC cells [24, 25]. Low bisecting GlcNAc levels on the surface of EVs promoted the carcinogenesis and metastasis of BC cells, while high bisecting GlcNAc levels can inhibit EVs-mediated metastasis [26]. To date, the effects of bisecting GlcNAc modifications on BC immunity and their regulatory mechanisms have not been thoroughly investigated.

In this study, we found that MGAT3 and the bisecting GlcNAc structure were closely associated with tumorigenesis and immune infiltration in BC. We demonstrated that bisecting GlcNAc modification could affect the function of PD-L1, and elucidated the detailed mechanisms by which this modification influenced PD-L1 and regulated T cell-mediated killing.

## Methods

### Bioinformatics analysis

The unified and standardized pan-cancer dataset were downloaded from the UCSC (https://genome.ucsc.edu/) database, and the N-glycan synthesis-related gene sets were obtained from the molecular signatures database (MSigDB, https://www.gsea-msigdb.org/gsea/msigdb/index.jsp). Differences in the expression of gene sets in cancer and normal tissues were analyzed using R software (version 4.2.1), the wilcoxon test was used for significance of differences analysis, and the “pheatmap” package was applied for visualization. Kaplan-Meier survival analysis and log-rank test were used to evaluate the survival difference between BC patients with high vs. low MGAT3 expression (grouped by median). Single-sample gene set enrichment analysis was performed using the “GSVA” and “GSEABase” packages, on the basis of which the ESTIMATE algorithm was used to assess immune score, stromal score, estimate score, and tumor purity [27]. Correlation of genes with immune infiltration analyzed online by Sangerbox (http://sangerbox.com/).

### Cell lines and cell culture

Human mammary epithelial cell line (MCF10A), human BC cell lines [MCF7, MDA-MB-231 (abbreviated as 231), MDA-MB-453, MDA-MB-468, BT549], and murine BC cell lines (4T1) were obtained from the Cell Bank of the Chinese Academy of Sciences (Shanghai, China). EO771 murine BC cells were purchased from Abcell (Beijing, China). MCF7, 231, MDA-MB-453, MDA-MB-468, and EO771 were cultured in DMEM medium containing 10% fetal bovine serum (FBS, Biological Industries; Be’er Sheva, Israel) and 1% penicillin/streptomycin (P/S). BT549 and 4T1 were cultured in RPMI 1640 containing 10% FBS and 1% P/S, respectively. MCF10A cells were grown in DMEM/F12 supplemented with 100 ng/ml cholera enterotoxin, 10 µg/ml recombinant human insulin, 0.5 µg/ml hydrocortisol, 20 ng/ml EGF, 5% horse serum, 100 UI/ml penicillin, and 100 µg/ml streptomycin. The above cells were cultured at 37℃ in 5% CO_2_ atmosphere.

### Western blotting and immunoprecipitation (IP)/ Co-immunoprecipitation (Co-IP)

Cells were washed with phosphate-buffered saline (PBS) and lysed in RIPA buffer (50 mM Tris, pH 7.2, 1% Triton X-100, 0.5% sodium deoxycholate, 0.1% SDS, 150 mM NaCl, 10 mM MgCl_2_, 5% glycerol) containing 1 µg/ml protease inhibitor at 4℃ for 30 min. Proteins were separated by sodium dodecyl sulphate-polyacrylamide gel electrophoresis (SDS-PAGE), and transferred onto PVDF membranes (Bio-Rad; CA, USA). The membranes were blocked with 3% bovine serum albumin (BSA, Beyotime Biotechnology; Jiangsu, China) in TBST at 37℃ for 1 h, incubated with primary antibodies overnight at 4℃, and then incubated with the corresponding HRP-labeled secondary antibodies at room temperature (RT) for 2 h. The target bands were visualized using the chemiluminescence image analysis system (Tanon Science & Technology Co.; Shanghai, China). For IP, cells were lysed as described above, and 500 µg of cell lysate was collected and incubated with 2 µg primary antibody at 4℃ for 2 h. A total of 20 µl protein A/G Plus-Agarose (Santa Cruz; CA, USA) was added, and mixed on rotation overnight at 4℃. The sample was washed, denatured with loading buffer, and subjected to western blotting analysis. All commercial antibodies were diluted and used according to the manufacturer’s instructions (Table S1).

### Immunohistochemistry (IHC)

Human BC and adjacent tissue samples were obtained from the Comprehensive Breast Care Center, the Second Affiliated Hospital of Xi’an Jiaotong University, and were approved by the Medical Ethics Committee of the Second Affiliated Hospital of Xi’an Jiaotong University. Written informed consent was obtained from all patients in accordance with the guidelines of the Declaration of Helsinki. Paraffin-embedded tissue sections were dewaxed, rehydrated, blocked with BSA, and then incubated with primary antibody at 4℃ overnight. After washing with PBS three times, the tissue was incubated with HRP-conjugated secondary antibody and visualized using 3,3’ -diaminobenzidine (DAB) staining reagent (MXB Biotechnology; Fujian, China). The signal intensity in tissue was calculated using Image Pro Plus (version 6.0, Media Cybernetics; CA, USA).

### Immunofluorescence

Cells were washed with PBS and fixed with 4% paraformaldehyde at RT for 15 min. Cells were blocked with 3% BSA at RT for 2 h and incubated with primary antibody PD-L1 (#66248-1-Ig, Proteintech; CA, USA) at 4℃ overnight. Subsequently, the cells were incubated with an Alexa Fluor 647-labeled anti-mouse secondary antibody (goat IgG, H + L; Beyotime) at RT for 1 h. After nuclear staining with 4′,6-diamidino-2-phenylindole (DAPI, Merck; Darmstadt, Germany), the fluorescence was visualized under confocal microscope (SP8, Leica; Wetzlar, Germany).

### RNA isolation and quantitative Real-Time PCR (qRT-PCR)

Total RNA was extracted using Trizol Reagent, and reversely transcribed to cDNA using ABScript III RT Master Mix (Abclonal; Wuhan, China). QRT-PCR as performed using Genious 2×SYBR Green Fast qPCR Mix (Abclonal). The gene expression was calculated according to the expression of GAPDH gene by 2^−ΔΔCt^ method [28]. The qRT-PCR primer sequences used are as follows: 5’-AACGGATTTGGTCGTATTG-3’ (GAPDH forward), 5’-GGAAGATGGTGATGGGATT-3’ (GAPDH reverse); 5’-TGGCATTTGCTGAACGCATTT-3’ (PD-L1 forward), 5’-TGCAGCCAGGTCTAATTGTTTT-3’ (PD-L1 reverse).

### Enzyme-linked immunosorbent assay (ELISA)

The 96-well ELISA plates (Jet Biofil; Guangzhou, China) were coated with the cell supernatant or patient plasma samples, incubated at 37 °C for 2 h, blocked with 3% BSA at RT for 1 h and washed with PBS. The primary antibody was added and incubated at 4 °C overnight. Plates were washed and incubated with HRP-labeled secondary antibody at 37 °C for 2 h. TMB solution (Beyotime) was added and incubated in dark at RT for 30 min, and 2 M sulfuric acid was added to terminate the reaction. The absorbance was measured at 450 nm with a microplate reader.

### Extraction and characteristic of EVs

Cells were cultured in DMEM medium containing 10% EVs-free FBS for 48 h. Supernatants were collected and centrifuged serially at 4℃: 500 g for 10 min, 2,000 g for 20 min, 10,000 g for 30 min, and 110,000 g for 70 min. EVs were rinsed with PBS and collected again for 70 min by ultracentrifugation at 100,000 g (Optima XE-100, Beckman Coulter Life Sciences; IN, USA) and resuspended in 100 µl PBS. To purify EVs from BC patient plasma, the plasma was centrifuged continuously at 4 °C, 3000 rpm for 15 min, 2,000 g for 20 min, and 10,000 g for 20 min, discard the supernatant and resuspend the pellet in 100 µl PBS. The separated EVs were placed on a 400-mesh carbon-coated grid (Electron Microscopy Sciences; PA, USA), stained with 2% uranyl acetate. Images were taken using a transmission electron microscope (TEM, H-7650, Hitachi; Tokyo, Japan). Dynamic Light Scattering (DLS) of EVs was performed using the Zetasizer high-sensitivity nanoparticle size analyzer (Malvern; UK).

### EVs internalization

EVs were labeled with ExoTracker, as described previously [29]. The labeled and unlabeled EVs were separately added to CD8^+^ T cells, incubated at 37℃ in dark for 30 min, and washed with PBS. The internalization of EVs were detected by flow cytometry (ACEA Biosciences; CA, USA).

### Isolation and culture of CD8^+^ T cells

Human-derived peripheral blood mononuclear cells (PBMCs) from venous blood and murine-derived PBMCs from spleen were isolated using human or murine lymphocyte isolation solution (Dakewe Biotech Co., Ltd.; Shenzhen, China), respectively. The corresponding CD8^+^ T cells were then isolated from PBMCs using MojoSort™ Human CD8 Nanobeads or MojoSort™ or Mouse CD8 T Cell Isolation Kit (BioLegend; CA, USA), respectively. Human CD8^+^ T cells were cultured in IMDM (Dakewe) with the addition of 10% Cell-Vive™ T Cell CD Serum Substitute (Biolegend), 100 IU/ml IL-2 (Novoprotein; Suzhou, China). Mouse CD8^+^ T cells were cultured in RPMI 1640 with the addition of 10% FBS and 100 IU/ml IL-2 (Novoprotein).

### In vitro T cell killing assay

CD8^+^ T cells were activated by PMA and ionomycin (MCE; NJ, USA) [30]. BC cells in logarithmic growth phase were treated with 5 µM carboxyfluorescein succinimidyl ester (CFSE; MCE), and incubated in dark at 37℃ for 1 h. After labeling, the cells were washed three times with PBS. The labeled BC cells were co-cultured with activated CD8^+^ T cells for 48 h. The apoptotic BC cells were stained with APC Annexin V (Biolegend) and 7-AAD (Biolegend), and detected by flow cytometry.

### IP/mass spectrometry (MS) analysis

PD-L1 protein was enriched by IP assay as described above. Briefly, pellets were separated by SDS-PAGE, stained by coomassie brilliant blue, decolorized in decolorization solution (30% acetonitrile, 50 mM NH_4_HCO_3_), denatured by 10 mM dithiothreitol and 20 mM iodoacetamide. The proteins were digested by trypsin (Promega; WI, USA) overnight at 37℃, and the peptides were collected. The glycopeptides were analyzed using QE HF-X (Thermofisher Scientific), and identified using the Glyco-Decipher software (version 1.0.4) [31].

### Plasmids and lentivirus infection

The murine MGAT3 was amplified via PCR and subsequently cloned into the lentiviral vector pLVX-Hygro (Takara; Shiga, Japan). The obtained pLVX-Hygro-MGAT3 (Forward: 5’-TCGTCTAGAATGAAGATGAGACGCTACAAGCTCTCT-3’, Reverse: 5’-GGGACCGGTGCCCTCCACTGTATCCAACTTG-3’) along with pMD2.G and psPAX2 (Addgene; MA, USA) was co-transfected into HEK293T. After 48 h, lentivirus particles were collected and transfected into 4T1 cells. The MGAT3 overexpressed cells 4T1M3 were selected using hygromycin and confirmed by western blotting. Similarly, the pLVX-OVA-Luc plasmid (Abiowell, Hunan, China) was applied for lentiviral packaging. The collected lentivirus was transfected into 4T1, 4T1M3, and EO771 cells. OVA overexpressing cells (4T1^OVA^, 4T1M3^OVA^, and EO771^OVA^) were screened by hygromycin and verified by flow cytometry. Human overexpressed MGAT3 and shMGAT3 plasmids were derived from previous studies [25].

### Construction of PD-L1 mutants

Human full-length PD-L1 was amplified by PCR from 231 cells. Site-directed mutagenesis was performed by fusion PCR. Mutants cells including 231-MGAT3-PD-L1^WT^, 231-MGAT3-PD-L1^N192Q^, 231-MGAT3-PD-L1^N200Q^, 231-MGAT3-PD-L1^N204Q^, and 231-MGAT3-PD-L1^N219Q^ (abbreviated 231M3^PDL1,^ 231M3^N192Q^, 231M3^N200Q^, 231M3^N204Q^, 231M3^N219Q^, respectively) were obtained by stably transfecting ransfecting pLVX plasmids containing Flag-tagged wild-type and site-mutant PD-L1 into 231-MGAT3 cells. Mutants cells including MCF7-PD-L1^WT^ and MCF7-PD-L1^N219Q^ (abbreviated MCF7^PDL1^ and MCF7^N219Q^, respectively) were obtained by stably transfecting pLVX plasmids containing Flag-tagged wild-type and site-mutant PD-L1 into MCF7 cells. The primer sequence for mutation were shown in Table S2.

### PD-L1 and PD-1 binding assay

To detect the interaction between PD-1 and PD-L1, BC cells were fixed in 4% paraformaldehyde for 15 min and incubated with recombinant human PD-1 protein (ABclonal) for 2 h, followed by incubation with Alexa Fluor 488 conjugated secondary antibody (Aladdin; Shanghai, China) at RT for 1 h. After resuspension in PBS, the cell-bound PD-1 was quantitatively analyzed by flow cytometry. To visualize PD-1 binding on the surface of BC cells, cells were incubated with Alexa Fluor 488 dye-bound PD-1 protein and observed using confocal microscope.

### Animal experiments

The implementation of the mice experiments was approved by the Biomedical Ethics Committee of the Medical Department of Xi’an Jiaotong University. For the subcutaneous xenograft experiment, 2 × 10^5^ 4T1^OVA^ or 4T1M3^OVA^ cells were injected subcutaneously in 5-week-old female BALB/c nude mice. Once tumors reached approximately 100 mm^3^, the OT-1 mouse (Model Organisms Center; Shanghai, China) derived CD8^+^ T^OT−1^ cells were injected intravenously into mice once every 3 days. For the subcutaneous homograft experiment, 5 × 10^5^ 4T1 cells were subcutaneously injected into 5-week-old female BALB/c mice, and 5 × 10^5^ EO771 cells were subcutaneously injected into 5-week-old female C57BL/6 mice. When the tumor grew to approximately 100 mm³, 5 mg/kg forskolin (MCE) [32] or anti-PD-L1 mAb (BioXCell, CA, USA) at 100 µg per mouse was injected intraperitoneally into mice once every 3 days. The control group was injected with IgG isotype control (Beyotime). B cells and Tregs were depleted via intraperitoneal injection of anti-CD20 (BioXCell) and anti-CD25 (BioXCell) antibodies (200 µg per mouse), respectively. Three days later, BALB/c mice were inoculated with 5 × 10⁵ 4T1 or 4T1M3 cells to monitor tumor growth. The control group was injected with IgG isotype control (Beyotime). Tumor size was measured every 3 days. Tumor weight was recorded and tumor volume was estimated based on 1/2× length× width^2^. At the end of the treatment course, mice were euthanized, tumors were excised, fixed, and subjected to IHC analysis.

### Statistical analysis

Statistical analysis was performed using GraphPad Prism 8 software. Data in bar graphs represent the multiplicative change in mean (± standard deviation) of three independent experiments. Normally distributed measures were analyzed using t-tests. One-way ANOVA was used for more than two groups. Differences were considered statistically significant at *P* < 0.05. *, *P* < 0.05; **, *P* < 0.01; ***, *P* < 0.001.

## Results

### MGAT3 as a key factor in the tumorigenesis and immune infiltration of BC

**Fig. 1:**
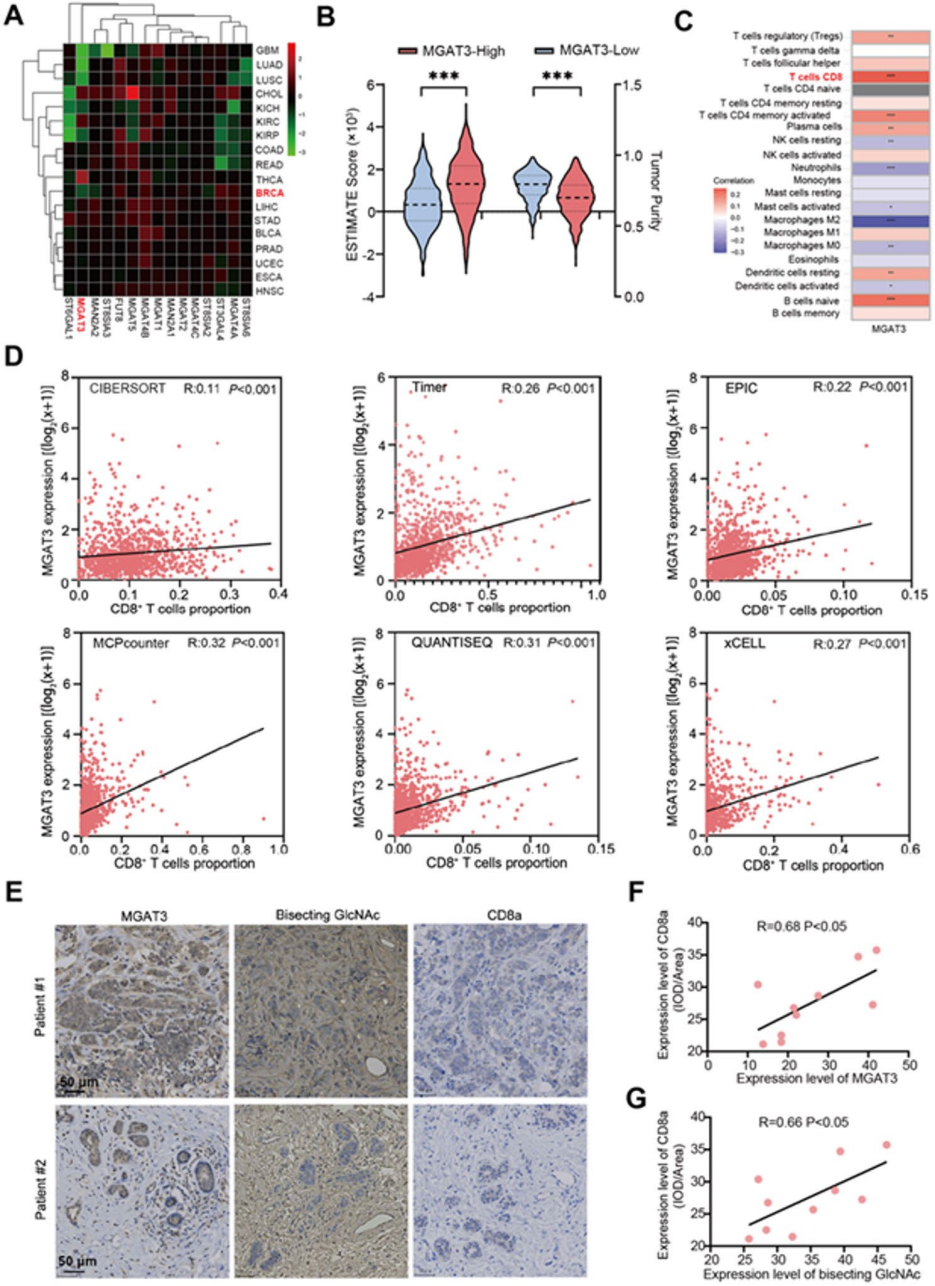
Identification of MGAT3 as a key factor in the tumorigenesis and immune infiltration of BC. **(A)** Bioinformatics were used to analyze the expression of 15 common glycosyltransferases in BC. **(B)** Differences in Estimate score and tumor purity between high- and low-MGAT3 samples. **(C)** Heatmap of the correlation between MGAT3 and BC infiltrating immune cells analyzed by the ssGSEA algorithm. **(D)** CIBERSORT, Timer, EPIC, MCPcounter, QUANTISEQ, and xCell algorithms were used to analyze the relationship between MGAT3 expression and CD8 T cell infiltration. **(E)** Representative IHC images of human BC tissue samples (left panel) and correlation of **(F)** MGAT3 and **(G)** bisecting GlcNAc with CD8 T cell infiltration (right panel)

Previous studies have demonstrated the involvement of aberrant N-glycosylation in the malignant progression of BC [33, 34]. Bioinformatic analysis revealed that β1,4-N-acetylglucosaminyltransferase III (MGAT3), which catalyzes the formation of bisecting GlcNAc (Fig. S1A), was significantly down-regulated in BC samples from the TCGA database (Fig. 1A). BC samples with high MGAT3 expression exhibited a significantly better overall survival compared to those with low MGAT3 expression (Fig. S1B). The ESTIMATE score, which assesses the composition of stromal and immune cells in malignant tumor tissues and indicates tumor purity [27], was significantly higher in the MGAT3 highly expressed group, vice versa, MGAT3 high expression was associated with lower tumor purity compared to the low-expression group (Fig. 1B). Immune infiltration analysis showed that CD8 T cells were more significantly enriched in BC with high expression of MGAT3 (Fig. 1C). CIBERSORT, TIMER, EPIC, MCPcounter, QUANTISEQ, xCell, and other recently developed immune infiltration algorithms have been used to enhance TME analysis and reduce inaccuracies and biases from relying on a single method [35], with these six algorithms further confirming that MGAT3 expression was positively correlated with the proportion of CD8 T cells (Fig. 1D). Stratified analysis of molecular subtypes of BC showed that MGAT3 expression level was positively correlated with CD8^+^ T cell infiltration in all BC subtypes (Fig. S1C-F). In contrast to normal breast tissues, the levels of MGAT3 and the bisecting GlcNAc were significantly reduced in BC tissues (Fig. S1G-J). IHC results also demonstrated that the level of MGAT3 and bisecting GlcNAc were positively related to the proportion of CD8^+^ T cells in BC tissues (Fig. 1E-G). BC cell lines, including 231, MCF7, MDA-MB-453, MDA-MB-468, and BT549, presented a significant decrease in MGAT3 expression in compared to normal breast epithelial cell line MCF10A (Fig. S1K).

### Bisecting GlcNAc levels in BC cells affects the killing efficacy of CD8^+^ T cells

**Fig. 2:**
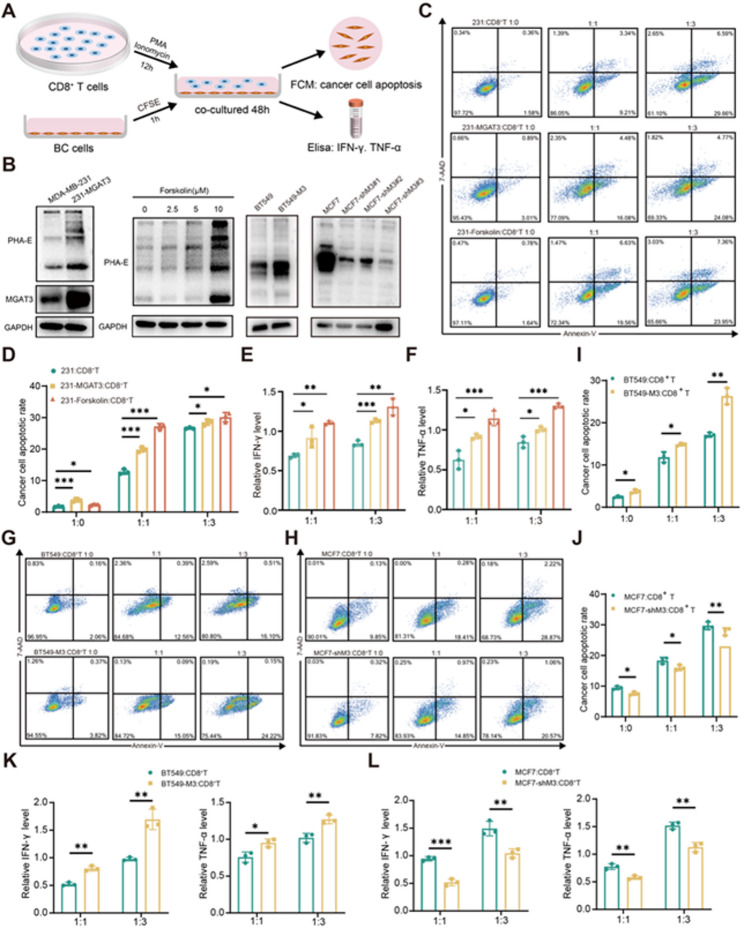
Bisecting GlcNAc levels in BC cells affects the killing efficacy of CD8^+^ T cells. **(A)** The flow chart of human CD8^+^ T cell activation and co-culture with BC cells in vitro. **(B)** The level of bisecting GlcNAc in BC cells was detected after overexpression of MGAT3 or forskolin treatment or knockdown of MGAT3. **C&D.** Flow cytometry was used to detect the apoptosis rates of 231/ 231-MGAT3/ 231-forskolin co-cultured with CD8^+^ T cells. **E&F.** Levels of IFN-γ and TNF-α secreted by CD8^+^ T cells in co-culture medium were measured by ELISA. Flow cytometry was used to detect the apoptosis rates of BT549/ BT549-M3 **(G&I)** and MCF7/ MCF7-shM3 **(H&J)** co-cultured with CD8^+^ T cells. Levels of IFN-γ and TNF-α in co-culture medium of **(K)** BT549/ BT549-M3 **(L)** MCF7/ MCF7-shM3 and CD8^+^ T cells were measured by ELISA

In view of the positive correlation between MGAT3 expression and the proportion of CD8^+^ T cells, we investigated whether the levels of bisecting GlcNAc in BC cells would impact the killing efficiency of CD8^+^ T cells. BC cells with different levels of bisecting GlcNAc were co-cultured with CD8^+^ T cells (Fig. 2A&B, S2A&B). The 231 cells with elevated levels of bisecting GlcNAc by MGAT3 overexpression (231-MGAT4) or forskolin treatment (an adenylyl cyclase activator that increases bisecting GlcNAc levels, referred as 231-Forskolin), which exhibited higher rates of cell apoptosis compared to untreated cells (Fig. 2C&D), accompanied by an elevation in the levels of effector cytokines IFN-γ and TNF-α of CD8^+^ T cells in the co-culture system (Fig. 2E&F). To investigate the dose-dependent response of cytotoxicity, we established co-culture systems with varying ratios of CD8⁺ T and tumor cells (effector-to-target, E:T ratios). With the increase of the proportion of CD8 + T cells incorporated in the co-culture system, the apoptosis rate of BC cells simultaneously increased. Consistent with these findings, BT549 cells overexpressing MGAT3 (BT549-M3) exhibited significantly higher levels of apoptosis when co-cultured with CD8^+^ T cells (Fig. 2G&I), accompanied by elevated levels of IFN-γ and TNF-α (Fig. 2K). Conversely, in co-culture systems of MGAT3-knockdown MCF7 (MCF7-shM3) and CD8^+^ T cells, both apoptosis rates and IFN-γ/TNF-α were markedly reduced (Fig. 2H&J&L). Collectively, these data suggested that higher levels of bisecting GlcNAc significantly enhanced the sensitivity of BC cells to CD8^+^ T cell killing.

### Bisecting GlcNAc promotes lysosomal degradation of PD-L1

**Fig. 3:**
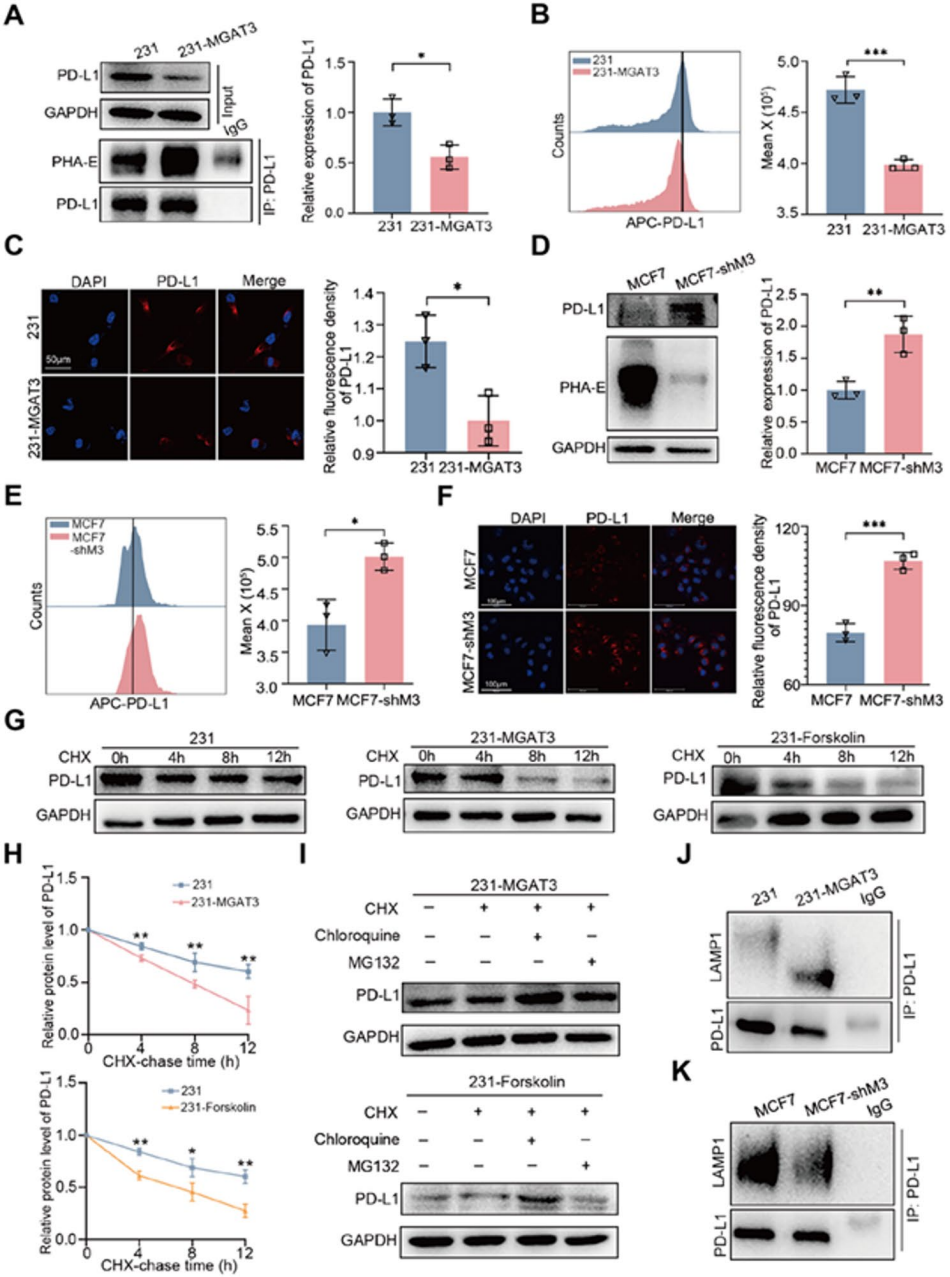
Bisecting GlcNAc promotes lysosomal degradation of PD-L1. Expression of PD-L1 protein in 231 and 231-MGAT3 was analyzed by **(A)** western blot, **(B)** flow cytometry and **(C)** confocal microscope. Expression of PD-L1 protein in MCF7 and MCF7-shM3 was analysed by **(D)** western blot, **(E)** flow cytometry and **(F)** confocal microscope. **G&H.** CHX (20 µM) chase analysis of PD-L1 expression in 231, 231-MGAT3 and 231-Forskolin cells. **I.** PD-L1 expression in 231-MGAT3 and 231-Forskolin cells treated with chloroquine or MG132. **J.** The interaction between PD-L1 and LAMP1 was detected by co-IP in 231 and 231-MGAT3. **K.** The interaction between PD-L1 and LAMP1 was detected by co-IP in MCF7 and MCF7-shM3

It is well known that the interaction between PD-L1 on the surface of tumor cells and PD-1 on CD8^+^ T cells plays a crucial role in determining T cell-mediated anti-tumor activity [36]. Building on the fact that PD-L1 is a typical glycoprotein found on the cell membrane, we hypothesized that bisecting GlcNAc modification may regulate the killing function of CD8^+^ T cells by affecting the expression of PD-L1 in BC. Notably, PD-L1 expression was not altered at mRNA level (Fig. S3A). At the protein level, IP assays confirmed the presence of bisecting GlcNAc modifications on PD-L1, with significantly enriched bisecting GlcNAc modifications observed on PD-L1 proteins in 231-MGAT3, 4T1M3, and BT549-M3 cells compared to their wild-type counterparts (Fig. 3A, S3B&C). Flow cytometry and immunofluorescence further confirmed that increasing the bisecting GlcNAc level significantly reduced PD-L1 expression (Fig. 3B&C). Consistently, PD-L1 level was significantly higher in MCF7-shM3, which stably knocks down MGAT3 via shRNA, compared to that in MCF7 (Fig. 3D-F). In BC tissue samples, we also observed a significant negative correlation between bisecting GlcNAc and PD-L1 (Fig. S3D). Through cycloheximide (CHX, a protein synthesis inhibitor) chase analysis, we found that increasing bisecting GlcNAc modification shortened the half-life of PD-L1 protein (Fig. 3G&H, S3E&G), while reducing bisecting GlcNAc modification prolonged its half-life (Fig. S3F&H). The expression of PD-L1 was significantly up-regulated in chloroquine (a lysosomal degradation inhibitor) treated 231-MGAT3, 231-Forskolin (Fig. 3I), BT549-M3 (Fig. S3I) and MCF7 (Fig. S3J) but not in MG132 (a proteasomal degradation inhibitor) treated cells. The interaction between PD-L1 and a lysosomal marker, LAMP1 [37] was enhanced in 231-MGAT3 (Fig. 3J) while weakened in MCF7-shM3 (Fig. 3K), indicating that the bisecting GlcNAc modification reduced PD-L1 expression by promoting the degradation via lysosomal pathway.

### The bisecting GlcNAc modification in BC EVs indirectly affects the killing efficiency of CD8^+^ T cells

**Fig. 4:**
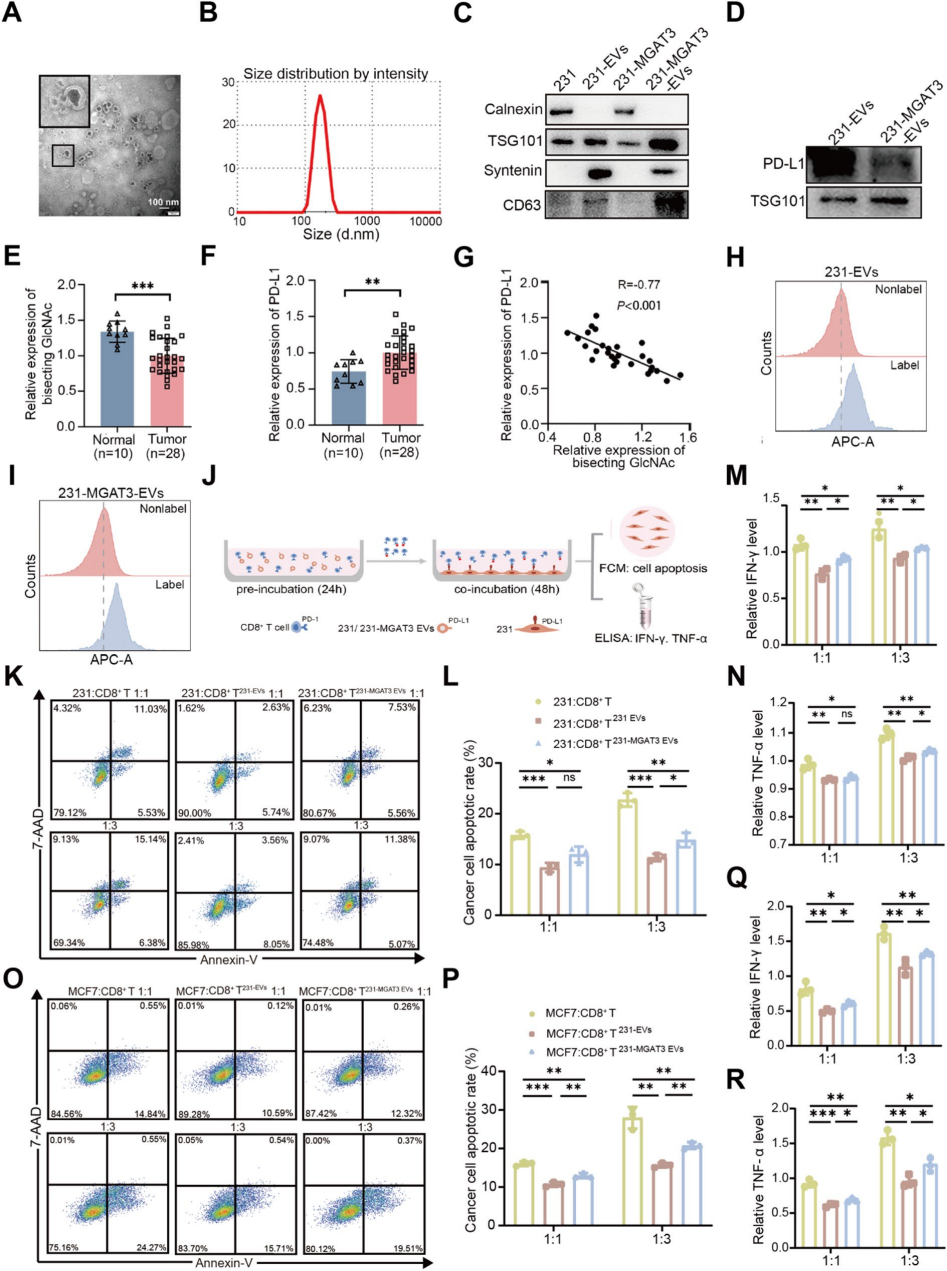
The bisecting GlcNAc modification in BC EVs indirectly affects killing efficiency of CD8^+^ T cells. **(A)** TEM images of EVs isolated from 231 cells. **(B)** DLS analysis. **(C)** Expression of EVs markers in 231-EVs and 231-MGAT3-EVs. **(D)** PD-L1 expression in 231-EVs and 231-MGAT3-EVs was analyzed by western blot. The relative expression levels of **(E)** bisecting GlcNAc and **(F)** PD-L1 in plasma EVs of normal people and BC patients were detected by ELISA. **G.** The correlation between bisecting GlcNAc and PD-L1 levels in plasma EVs of BC patients was detected by ELISA. **H&I.** Flow cytometry was used to analyze the internalize of Exotracker labeled EVs by CD8^+^ T cells. **J.** Flowchart of co-culture of CD8^+^ T with 231 cells in vitro after pre-incubating with 231-EVs or 231-MGAT3-EVs. Flow cytometry was used to detect the apoptosis rates of **(K&L)** 231 and **(O&P)** MCF7 cells. Levels of IFN-γ and TNF-α in co-culture medium of **(M&N)** 231 or **(Q&R)** MCF7 and CD8^+^ T cells were measured by ELISA

Similar to PD-L1 on the cell membrane, PD-L1 on EVs also plays a role in suppressing immune activity [38]. In this study, we isolated EVs from 231 and 231-MGAT3 cells (231-EVs and 231-MGAT3-EVs), exhibiting spherical morphology (Fig. 4A), a size of ~ 150 nm (Fig. 4B), and expression of EV markers TSG101, Syntenin, and CD63 (Fig. 4C), meeting the criteria set by the International Society for Extracellular Vesicles [39]. The expression of PD-L1 was significantly lower in 231-MGAT3-EVs than that in 231-EVs (Fig. 4D). In plasma EVs of BC patients, low levels of bisecting GlcNAc (Fig. 4E) and higher levels of PD-L1 (Fig. 4F) were observed, which presenting negatively correlation (Fig. 4G). 231-EVs and 231-MGAT3-EVs could be effectively internalized by CD8^+^ T cells (Fig. 4H&I). When CD8^+^ T cells were pre-incubated with EVs containing low levels of bisecting GlcNAc (231-EVs) and then co-cultured with 231 cells for 48 h, their cytotoxic capacity was reduced compared to untreated CD8^+^ T cells, indicating that low bisecting GlcNAc induced immunosuppression (Fig. 4J). However, treatment with high levels of bisecting GlcNAc (231-MGAT3-EVs) significantly attenuated this immunosuppressive effect (Fig. 4K&L). The consistent trend of IFN-γ and TNF-α levels secreted by 231-EVs or 231-MGAT3-EVs treated CD8^+^ T cells was observed (Fig. 4M&N). At the same time, pre-treatment of CD8^+^ T cells with 231-EVs also suppressed their ability to kill MCF7 cells relative to untreated CD8^+^ T cells, accompanied by a decrease secretion of IFN-γ and TNF-α, whereas 231-MGAT3-EVs reversed this immunosuppressive effect, enhancing MCF7 cells apoptosis and the release of these cytokines (Fig. 4O-R). These results demonstrated that EVs with high levels of bisecting GlcNAc exhibited lower PD-L1 levels, which could indirectly enhance the killing efficiency of CD8^+^ T cells.

### The killing efficiency of CD8^+^ T cells in vivo

**Fig. 5:**
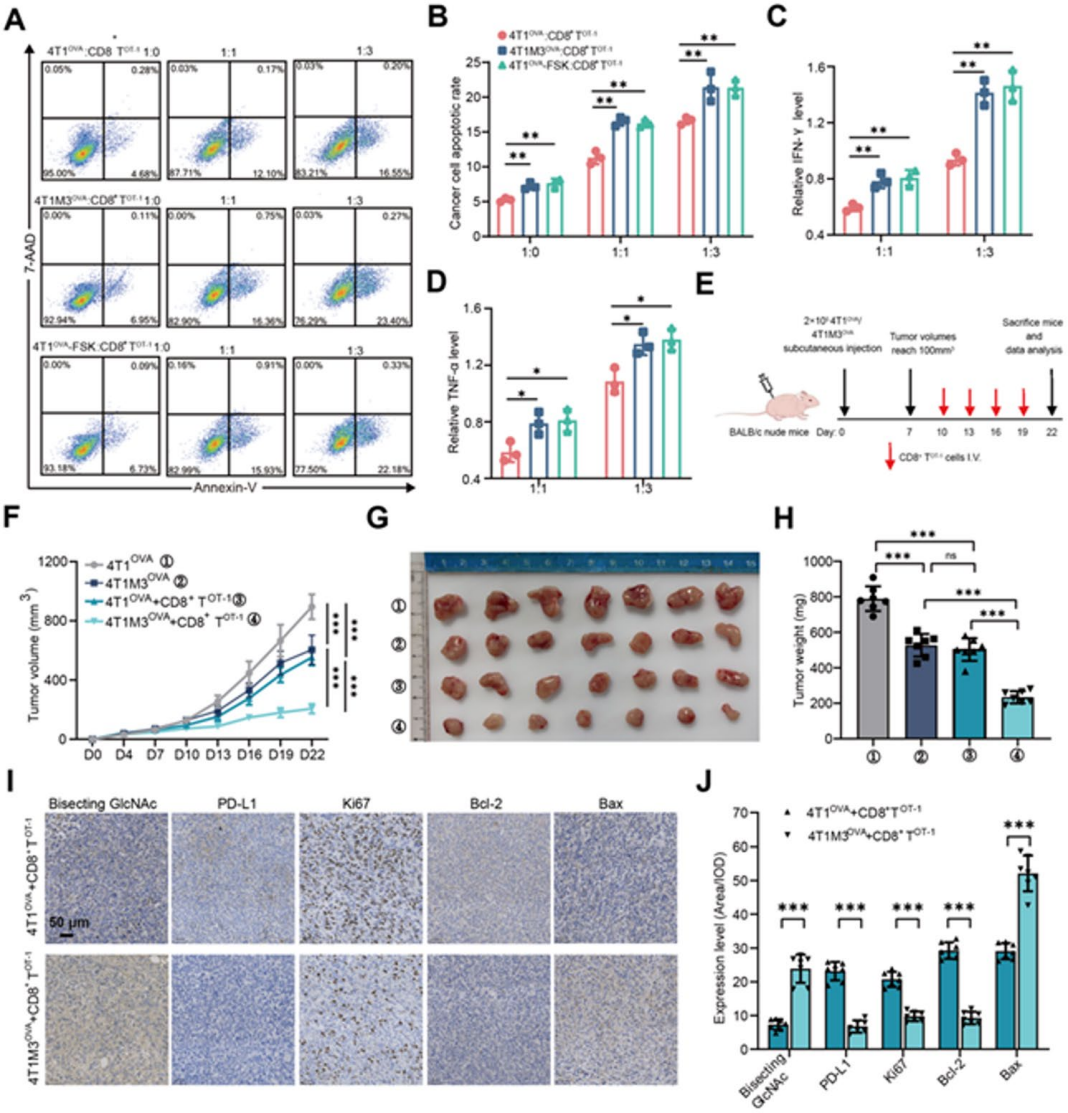
The killing efficiency of CD8^+^ T cells in vivo. **A&B.** Flow cytometry was used to detect the apoptosis rates of 4T1^OVA^, 4T1M3^OVA^, and 4T1^OVA^-FSK cells. **C&D.** Levels of IFN-γ and TNF-α in co-culture medium were measured by ELISA. **E.** Flowchart of CD8^+^ T^OT−1^ cell-mediated killing of BC cells in vivo. **F.** Tumor growth curves. **G.** Tumor from BALB/c female nude mice bearing 4T1^OVA^/4T1M3^OVA^ cells and treated with or without CD8^+^ T^OT−1^ cells. **H.** Tumors weight. **I&J.** Representative IHC images and comparison of bisecting GlcNAc, PD-L1, Ki67, Bcl-2, and Bax levels

We established tumor models (4T1M3, 4T1-FSK, and EO771-FSK) with elevated bisecting GlcNAc levels through MGAT3 overexpression or forskolin treatment (Fig. S4A&B). To further assess antigen-specific killing, we extracted CD8^+^ T^OT−1^ cells from OT-1 mice (Fig. S4C), and further constructed 4T1^OVA^ (Fig. S4D), 4T1M3^OVA^ (Fig. S4E), EO771^OVA^ (Fig. S4F), which express high level of model antigen ovalbumin (OVA257-264) that can be specifically recognized by CD8^+^ T^OT−1^ cells [40]. Then CD8^+^ T^OT−1^ was co-cultured with OVA-expression murine tumor cells (Fig. S4G). Compared to 4T1^OVA^ cells, CD8^+^ T^OT−1^ cells demonstrated enhanced cytotoxicity against 4T1M3^OVA^ and 4T1^OVA^-FSK, as evidenced by higher tumor cell apoptosis rates (Fig. 5A&B) and elevated secretion of effector cytokines (IFN-γ and TNF-α) in co-culture systems (Fig. 5C&D). This bisecting GlcNAc-dependent immunostimulatory phenotype was consistently observed in EO771^OVA^ cells (Fig. S4H&I), where EO771^OVA^-FSK cells exhibited superior susceptibility to CD8^+^ T^OT−1^ cell-mediated killing with parallel increases in IFN-γ and TNF-α levels (Fig. S4J&K). When 4T1^OVA^ and 4T1M3^OVA^ cells were subcutaneously implanted into nude mice, followed by intravenous injection of CD8^+^ T^OT−1^ cells, tumor growth was significantly slower in 4T1M3^OVA^ tumor-bearing mice compared to those bearing 4T1^OVA^ tumors (Fig. 5E&F). CD8^+^ T^OT−1^ effectively reduced the tumor burden in 4T1M3^OVA^ tumor-bearing mice (Fig. 5G&H). IHC staining revealed that in 4T1M3^OVA^ tumors, the levels of bisecting GlcNAc and pro-apoptotic protein Bax were increased, and the levels of PD-L1, cell proliferation index Ki67 and anti-apoptotic protein Bcl-2 were decreased, indicating that CD8^+^ T^OT−1^ cells promoted the apoptosis of BC cells with high bisecting GlcNAc levels (Fig. 5I&J). The 4T1M3^OVA^ group excluded intrinsic tumor interference, as differences of Bax/ Bcl-2/ Ki67 compared with CD8⁺ T^OT−1^-treated 4T1M3^OVA^ tumors were significantly smaller than those between the two CD8⁺ T^OT−1^-treated tumor groups, demonstrating that these changes of these indicators were due to the different cytotoxic effects of CD8⁺ T^OT−1^ cells (Fig. S4L&M). The above results confirmed that bisecting GlcNAc modification on BC cells enhanced the killing efficiency of CD8^+^ T cells by down-regulating the PD-L1 expression in vivo.

However, in mice with complete immune system, there are multiple immunoregulatory cells in the TME. To determine whether the antitumor effect of MGAT3 specifically depends on CD8⁺ T cells, we depleted Treg cells and B cells using anti-CD25 and anti-CD20 antibodies, respectively, in BALB/c mice. The impact of MGAT3 overexpression on tumor growth was subsequently evaluated (Fig. S5A). The results demonstrated that specific depletion of B cells/Tregs have no significant effects on tumor volume and weight in 4T1 or 4T1M3 bearing mice. Tumor growth was significantly suppressed the 4T1M3 group with/without depletion of B cells/Tregs compared to 4T1 group (Fig. S5B-D). These data further support that MGAT3 exerts antitumor effects mainly by specifically enhancing the efficacy of CD8^+^ T cells.

### The bisecting GlcNAc modification affects PD-L1 binding to PD-1

**Fig. 6:**
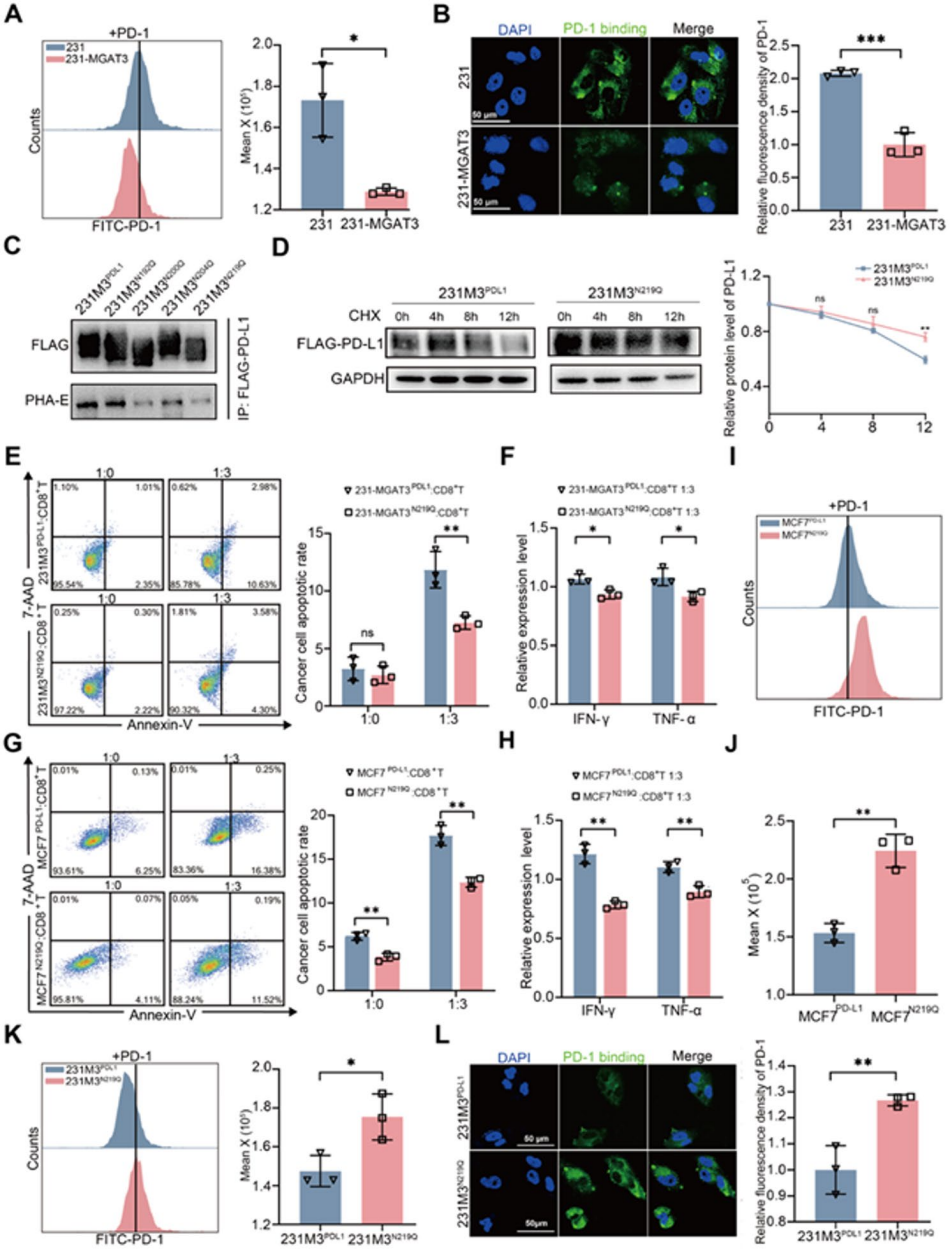
The bisecting GlcNAc modification affects PD-L1 binding to PD-1. The recombinant PD-1 binding to PD-L1 on the cell membrane of 231 and 231-MGAT3 was detected by **(A)** flow cytometry; and **(B)** confocal microscope. **C.** The levels of bisecting GlcNAc in PD-L1^WT^ and various PD-L1 mutants were detected by IP and western blot analysis. **D.** CHX (20 µM) chase analysis of PD-L1 expression in 231M3^PDL1^ and 231M3^N219Q^ cells. Flow cytometry was used to detect the apoptosis rates of **(E)** 231M3^PDL1^/ 231M3^N219Q^ or **(G)** MCF7^PDL1^/ MCF7^N219Q^ cells co-cultured with CD8^+^ T cells. IFN-γ and TNF-α secreted by CD8^+^ T cells in co-culture medium of **(F)** 231M3^PDL1^/ 231M3^N219Q^ or **(H)** MCF7^PDL1^/ MCF7^N219Q^ were measured by ELISA. **I&J.** The recombinant PD-1 binding to PD-L1 on the membrane of MCF7^PDL1^ and MCF7^N219Q^ cells was detected by flow cytometry. The recombinant PD-1 binding to PD-L1 on the membrane of 231M3^PDL1^ and 231M3^N219Q^ cells was detected by **(K)** flow cytometry and **(L)** confocal microscope

Previous studies have shown that the glycosylation of PD-L1 affects its immunosuppressive function [17]. When equivalent recombinant PD-1 was exogenously added, the binding of PD-1 to PD-L1 on MGAT3 overexpressing cells was significantly attenuated (Fig. 6A&B, S6A), whereas enhanced in shMGAT3 cells (Fig. S6B), indicating that bisecting GlcNAc may affect the interaction between PD-1 and PD-L1. With glycoproteomics technique, we identified four N-glycosylation sites, N192, N200, N204, and N219 in PD-L1 (Fig. S6C-F, Table S3). And these four asparagines (Asn) were separately replaced with glutamine (Gln) in 231-MGAT3, termed 231M3^N192Q^, 231M3^N200Q^, 231M3^N204Q^ and 231M3^N219Q^ cells. Compared with PD-L1^WT^, the level of bisecting GlcNAc was significantly decreased in PD-L1^N219Q^, revealing that N219 was the main bisecting GlcNAcylated site (Fig. 6C). The degradation rate of PD-L1 was slower in 231M3^N219Q^ than that in 231M3^PDL1^ (Fig. 6D). The T cell-mediated tumor cell killing assay showed that compared with 231M3^PDL1^, the apoptosis rate of 231M3^N219Q^ was significantly inhibited (Fig. 6E), accompanied by the decrease of effector cytokines IFN-γ and TNF-α secreted by CD8^+^ T cells in co-culture system (Fig. 6F). Similarly, compared to MCF7^PDL1^, MCF7^N219Q^ exhibited enhanced resistance to CD8^+^ T cell-mediated killing (Fig. 6G), accompanied by reduced levels of IFN-γ and TNF-α (Fig. 6H). Flow cytometry (Fig. 6I-K) and immunofluorescence analyses (Fig. 6L) collectively demonstrated that the binding of recombinant PD-1 to PD-L1^N219Q^ was significantly enhanced compared to PD-L1^WT^.

### Increasing bisecting GlcNAc level enhances the sensitivity of BC to anti-PD-L1 therapy

**Fig. 7:**
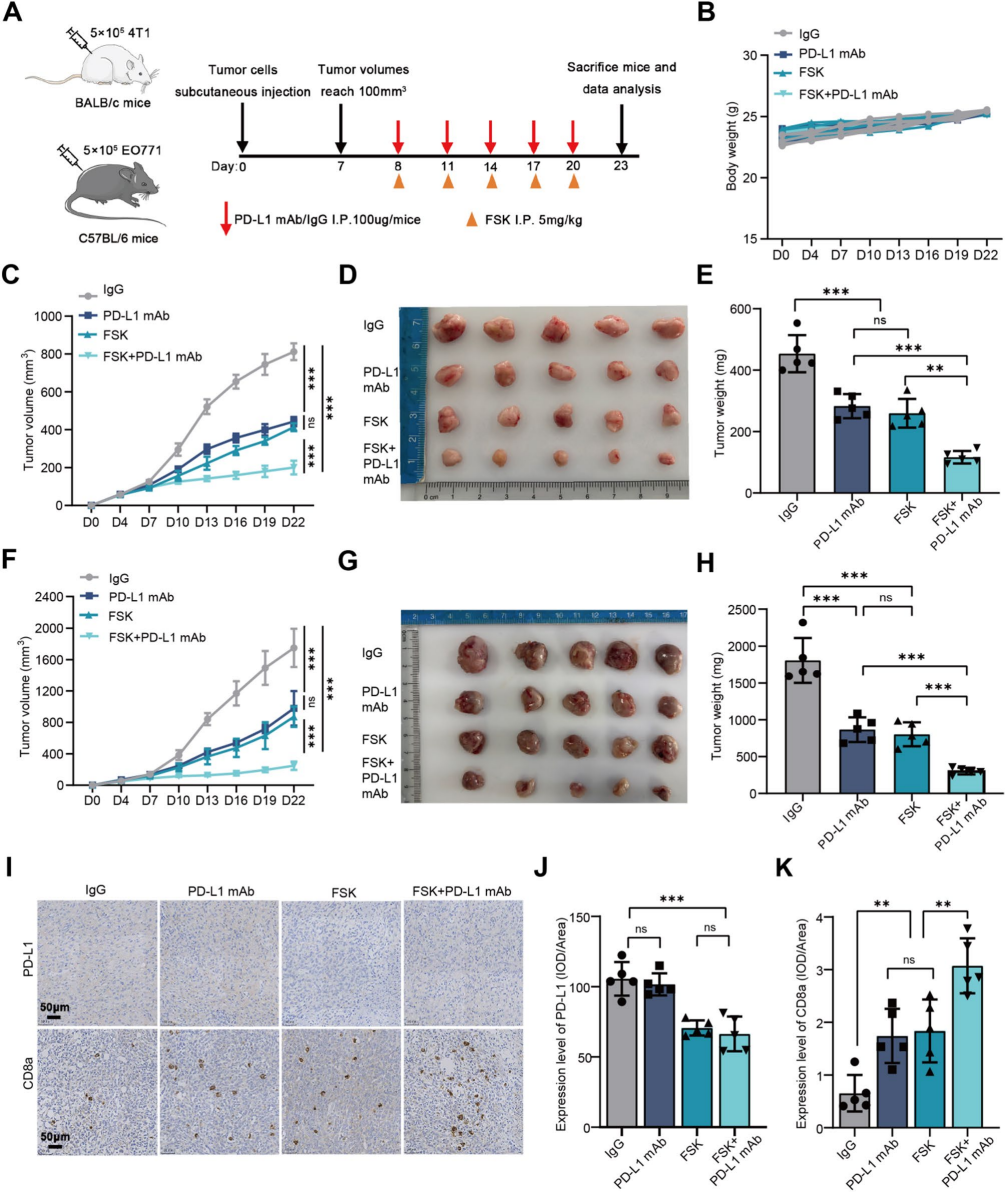
Increasing bisecting GlcNAc level enhances the sensitivity of BC to anti-PD-L1 therapy. **(A)** The schematic illustration of experimental design. **(B)** The body weight of 4T1 tumor-bearing mice in different groups. **(C)** Tumor growth curves, **(D)** tumor images, and **(E)** tumor weight in 4T1 tumor-bearing mice. **(F)** Tumor growth curves, **(G)** tumor images, and **(H)** tumor weight in EO771 tumor-bearing mice. **(I)** Representative IHC images and **(J)** comparison of PD-L1 expression and **(K)** infiltration proportion of CD8^+^ T cells of 4T1 tumor-bearing mice in different groups

To evaluate the regulatory effect of bisecting GlcNAc on immunotherapy in vivo (Fig. 7A), we constructed 4T1 and EO771 tumor-bearing mice and treated them with forskolin, alone or in combination with anti-PD-L1 monoclonal antibody (PD-L1 mAb ). Importantly, no significant weight loss or other common signs of toxicity were observed in the combination treatment group (Fig. 7B). Compared to the control group, forskolin treatment modestly delayed tumor growth, achieving a therapeutic effect comparable to that of PD-L1 mAb. Notably, the combination of forskolin and PD-L1 mAb significantly reduced the growth rate (Fig. 7C&F) and tumor burden of mice (Fig. 7DE&GH). IHC staining revealed that increasing bisecting GlcNAc levels by forskolin reduced PD-L1 expression while increasing the proportion of tumor-infiltrating CD8^+^ T cells. Moreover, the combined treatment demonstrated a significant synergistic effect in enhancing CD8^+^ T-cell infiltration (Fig. 7I-K). These findings confirm that increasing bisecting GlcNAc enhances CD8^+^ T-cell infiltration and function by downregulating PD-L1 expression, thereby improving the efficacy of PD-L1 mAb therapy.

## Discussion

The complex “glyco-code” formed by glycosylation on the cell surface has an important impact on tumor development and progression [19]. The most common abnormal glycosylations include truncated O-glycan, increased branching N-glycan, and changes in sialylation and fucosylation. Altered glycosylation of tumor cell surface proteins can help cancer cells evade immune detection, while targeting glycosylation improves tumor immunotherapy and provides new clinical perspectives for advancing tumor therapy [41]. For instance, the application of sialidase or sialoglycan synthesis inhibitors interferes with sialoglycan synthesis, promoting the immune control of tumors [42–44]. The knocking out MGAT5 or using 2-Deoxy-D-glucose in pancreatic adenocarcinoma inhibited the synthesis of branching N-glycans, resulting in enhanced activity and reduced exhaustion of CAR-T cells [45]. Our study demonstrated here that the levels of MGAT3 and the bisecting GlcNAc were significantly reduced in BC tissues, and bisecting GlcNAc levels were positively related to the proportion of CD8^+^ T cells in BC tissues.

As a special form of N-glycans, bisecting GlcNAc formation has been widely shown to prevent further extension of the dual-antenna N-glycan structures by other glycosyltransferases, including MGAT2, MGAT4, MGAT5, and FUT8 [46, 47]. Bisecting GlcNAc can modify membrane proteins such as integrins, growth factor receptors and ABC transporters, affecting their expression and subsequently influencing the signaling pathways in which these proteins are involved [48–50]. As a glycoprotein, the structure and biological function of PD-L1 are largely regulated by glycosylation status [51]. N-glycosylation on PD-L1 protein has been reported to promote T-cell immune escape by inhibiting glycogen synthase kinase 3β-mediated degradation of 26 S protease, which in turn serves to stabilize PD-L1 protein expression, maintain its interaction with PD-1 [17]. B4GALT1 mediated galactosylation promotes immune escape in lung adenocarcinoma by up-regulating PD-L1 expression and inhibiting CD8^+^ T cell infiltration [52].Here, we found that elevated bisecting GlcNAc levels in BC cells promoted PD-L1 degradation. Also, the modification by bisecting GlcNAc inhibits the binding of PD-L1 to PD-1, suggesting that specific glycan structures may spatially hinder their binding capacity. Together, these synergistic effects enhanced the ability of CD8^+^ T cells to eliminate tumor cells.

**Fig. 8:**
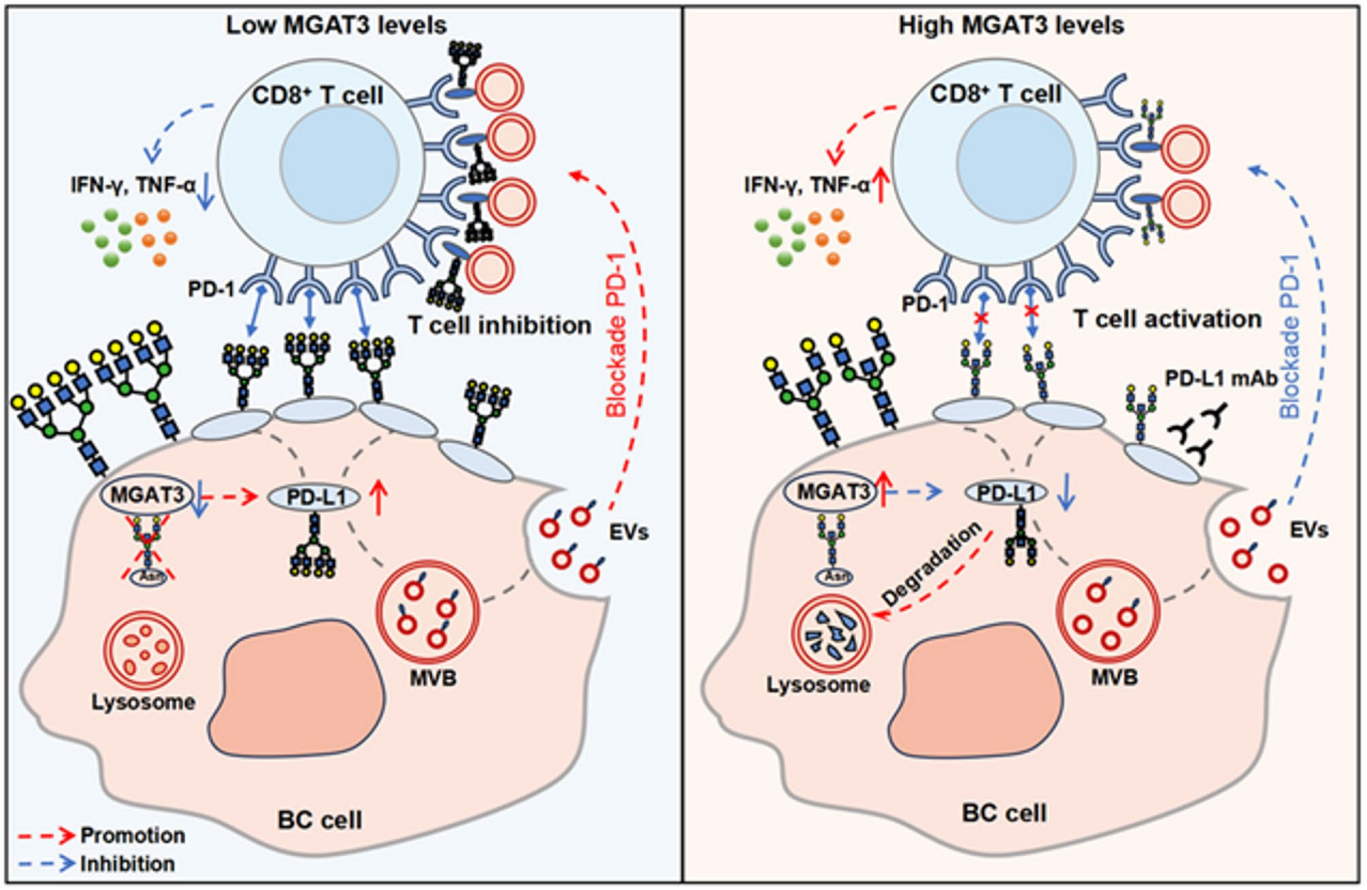
Bisecting GlcNAc modification regulates PD-L1 to affect CD8^+^ T cell killing in BC cells

In addition, EVs secreted by tumor cells present PD-L1 on their surface, which can suppress the immune response by blocking PD-1 on CD8^+^ cells [53]. EVs with high expression of PD-L1 in head and neck squamous cell carcinoma significantly inhibited CD69 on CD8^+^ T cells and inhibited T cell activation [54]. Stimulation with IFN-γ increases the amount of PD-L1 on EVs released by metastatic melanomas, which suppresses the function of CD8 T cells and facilitates tumour growth [55]. Pretreatment of glioblastoma EVs with anti-PD-1 antibody significantly reversed the inhibition of T cells and prevented tumor progression [56]. In this study, we found that EVs derived from high levels bisecting GlcNAc modification presented lower PD-L1 and enhanced the killing efficiency of CD8^+^ T cells (Fig. 8). Conversely, this also suggests that disrupting the blocking effect of EVs on CD8^+^ T cells could enhance their killing ability from another perspective.

Recently, the regulation of TME by targeting abnormal glycosylation has become one of the hotspots to improve the efficacy of tumor immunotherapy. The combination of OGT inhibitor OSMI-4 and PD-L1 mAb can restore tumor immunity and synergistically inhibit the growth of liver cancer and melanoma in fully immunized mice [23]. Therapeutic desialylation can polarize tumor-associated macrophages into immune-promoting phenotype, showing the potential to enhance the efficacy of ICIs [57]. Previous studies have shown that increasing MGAT3 expression can restore sensitivity to ICI therapy, offering a potential new strategy to overcome immune resistance in colorectal cancer [58]. Our in vivo experiments demonstrated that combining forskolin with PD-L1 mAb synergistically enhances anti-tumor activity. The elevation of bisecting GlcNAc levels accelerates PD-L1 degradation through the lysosomal pathway, thereby reducing its translocation to the cell membrane, diminishing the expression of PD-L1 on the cell surface, and inhibiting PD-L1/PD-1 interactions, while addition of PD-L1 mAb further neutralizes residual PD-L1 on the cell surface (Fig. 8). This combined approach increases the sensitivity of BC cells to PD-L1 mAb therapy and significantly boosts T-cell-mediated immune killing. Thus, therapeutically enhancing GlcNAc modification in combination with anti-PD-1/PD-L1 or other ICIs offers a promising strategy to improve T-cell immunoreactivity and strengthen the anti-tumor immune response in BC. It is worth noting that future research needs to systematically explore whether and how MGAT3-mediated bisecting GlcNAc alterations affect other crucial immune cell subsets in the TME, such as myeloid-derived suppressor cells, tumor-associated macrophages, regulatory T cells, and natural killer cells [59]. Elucidating the global regulatory role of MGAT3 in the TME will facilitate the development of more comprehensive combinatorial immunotherapy strategies.

## Conclusion

In summary, our study found that high bisecting GlcNAc modification not only promoted the PD-L1 degradation, but also inhibited the binding of PD-L1 to PD-1, thereby enhancing CD8^+^ T cell-mediated cytotoxicity, and improving the PD-L1 mAb sensitivity, presenting a certain theoretical basis for bisecting GlcNAc modification as a potential therapeutic target in BC.

## Supplementary Information

Below is the link to the electronic supplementary material.

Supplementary Material 1

Supplementary Material 2

Supplementary Material 3

Supplementary Material 4

## Data Availability

No datasets were generated or analysed during the current study.

## Abbreviations

BC: Breast cancer
TME: Tumor microenvironment
ICIs: Immune checkpoint inhibitors
mAbs: Monoclonal antibodies
EVs: Extracellular vesicles
PTM: Post-translational modification
MGAT3: β1,4-N-acetylglucosaminyltransferase III
FBS: Fetal bovine serum
PBS: Phosphate-buffered saline
BSA: Bovine serum albumin
RT: Room temperature
IP: Immunoprecipitation
IHC: Immunohistochemistry
qRT-PCR: Quantitative real-time PCR
ELISA: Enzyme-linked immunosorbent assay
TEM: Transmission electron microscope
DLS: Dynamic light scattering
PBMCs: Peripheral blood mononuclear cells
CFSE: Carboxyfluorescein succinimidyl ester
MS: Mass spectrometry

## References

[1] Siegel RL, Miller KD, Wagle NS, Jemal A. Cancer statistics, 2023. CA Cancer J Clin. 2023;73(1):17–48. 36633525 10.3322/caac.21763

[2] Li T, Zhang H, Lian M, He Q, Lv M, Zhai L, et al. Global status and attributable risk factors of breast, cervical, ovarian, and uterine cancers from 1990 to 2021. J Hematol Oncol. 2025;18(1):5. 39794860 10.1186/s13045-025-01660-y PMC11721161

[3] Zhu S, Wu Y, Song B, Yi M, Yan Y, Mei Q, et al. Recent advances in targeted strategies for triple-negative breast cancer. J Hematol Oncol. 2023;16(1):100. 37641116 10.1186/s13045-023-01497-3 PMC10464091

[4] Yang Y. Cancer immunotherapy: Harnessing the immune system to battle cancer. J Clin Invest. 2015;125(9):3335–7. 26325031 10.1172/JCI83871 PMC4588312

[5] Emens LA. Breast cancer immunotherapy: facts and hopes. Clin Cancer Res. 2018;24:511–20. 28801472 10.1158/1078-0432.CCR-16-3001 PMC5796849

[6] Vranic S, Cyprian FS, Gatalica Z, Palazzo J. PD-L1 status in breast cancer: current view and perspectives. Semin Cancer Biol. 2021;72:146–54. 31883913 10.1016/j.semcancer.2019.12.003

[7] Ma W, Xue R, Zhu Z, Farrukh H, Song W, Li T, et al. Increasing cure rates of solid tumors by immune checkpoint inhibitors. Exp Hematol Oncol. 2023;12(1):10. 36647169 10.1186/s40164-023-00372-8 PMC9843946

[8] Robbins PD, Morelli AE. Regulation of immune responses by extracellular vesicles. Nat Rev Immunol. 2014;14(3):195–208. 24566916 10.1038/nri3622 PMC4350779

[9] Zhu S, Li S, Yi M, Li N, Wu K. Roles of microvesicles in tumor progression and clinical applications. Int J Nanomed. 2021;16:7071–90. 10.2147/IJN.S325448 PMC8536885 34703228

[10] Muntasell A, Berger AC, Roche PA. T cell-induced secretion of MHC class II-peptide complexes on B cell exosomes. The EMBO journal. EMBO J. 2007;26(19):4263–72. 17805347 10.1038/sj.emboj.7601842 PMC2230838

[11] Raposo G, Nijman HW, Stoorvogel W, Liejendekker R, Harding CV, Melief CJ, et al. B lymphocytes secrete antigen-presenting vesicles. J Exp Med. 1996;183(3):1161–72. 8642258 10.1084/jem.183.3.1161 PMC2192324

[12] Qazi KR, Gehrmann U, Domange Jordö E, Karlsson MC, Gabrielsson S. Antigen-loaded exosomes alone induce Th1-type memory through a B-cell-dependent mechanism. Blood. 2009;113(12):2673–83. 19176319 10.1182/blood-2008-04-153536

[13] Segura E, Nicco C, Lombard B, Véron P, Raposo G, Batteux F, et al. ICAM-1 on exosomes from mature dendritic cells is critical for efficient Naive T-cell priming. Blood. 2005;106(1):216–23. 15790784 10.1182/blood-2005-01-0220

[14] Mallegol J, Van Niel G, Lebreton C, Lepelletier Y, Candalh C, Dugave C, et al. T84-intestinal epithelial exosomes bear MHC class ii/peptide complexes potentiating antigen presentation by dendritic cells. Gastroenterology. 2007;132(5):1866–76. 17484880 10.1053/j.gastro.2007.02.043

[15] Asao T, Tobias GC, Lucotti S, Jones DR, Matei I, Lyden D. Extracellular vesicles and particles as mediators of long-range communication in cancer: connecting biological function to clinical applications. Extracell Vesicles Circ Nucl Acids. 2023;4(3):461–85. 38707985 10.20517/evcna.2023.37 PMC11067132

[16] Xie F, Xu M, Lu J, Mao L, Wang S. The role of Exosomal PD-L1 in tumor progression and immunotherapy. Mol Cancer. 2019;18(1):146. 31647023 10.1186/s12943-019-1074-3 PMC6813045

[17] Li CW, Lim SO, Xia W, Lee HH, Chan LC, Kuo CW, et al. Glycosylation and stabilization of programmed death ligand-1 suppresses T-cell activity. Nat Commun. 2016;7:12632. 27572267 10.1038/ncomms12632 PMC5013604

[18] Wang R, He S, Long J, Wang Y, Jiang X, Chen M, et al. Emerging therapeutic frontiers in cancer: insights into posttranslational modifications of PD-1/PD-L1 and regulatory pathways. Exp Hematol Oncol. 2024;13(1):46. 38654302 10.1186/s40164-024-00515-5 PMC11040904

[19] Eichler J. Protein glycosylation. Curr Biol. 2019;29(7):R229–31. 30939300 10.1016/j.cub.2019.01.003

[20] Guo Y, Jia W, Yang J, Zhan X. Cancer glycomics offers potential biomarkers and therapeutic targets in the framework of 3P medicine. Front Endocrinol. 2022;13:970489. 10.3389/fendo.2022.970489 PMC9441633 36072925

[21] RodrÍguez E, Schetters STT, van Kooyk Y. The tumour glyco-code as a novel immune checkpoint for immunotherapy. Nat Rev Immunol. 2018;18(3):204–11. 29398707 10.1038/nri.2018.3

[22] Wang J, Manni M, Bärenwaldt A, Wieboldt R, Kirchhammer N, Ivanek R, et al. Siglec receptors modulate dendritic cell activation and antigen presentation to T cells in cancer. Front Cell Dev Biol. 2022;10:828916. 35309936 10.3389/fcell.2022.828916 PMC8927547

[23] Zhu Q, Wang H, Chai S, Xu L, Lin B, Yi W, et al. O-GlcNAcylation promotes tumor immune evasion by inhibiting PD-L1 lysosomal degradation. Proc Natl Acad Sci U S A. 2023;120(13):e2216796120. 36943877 10.1073/pnas.2216796120 PMC10068856

[24] Cheng L, Cao L, Wu Y, Xie W, Li J, Guan F, et al. Bisecting N-Acetylglucosamine on EGFR inhibits malignant phenotype of breast cancer via down-regulation of egfr/erk signaling. Front Oncol. 2020;10:929. 32612952 10.3389/fonc.2020.00929 PMC7308504

[25] Tan Z, Wang C, Li X, Guan F. Bisecting N-Acetylglucosamine structures inhibit Hypoxia-Induced Epithelial-Mesenchymal transition in breast cancer cells. Front Physiol. 2018;9:210. 29593568 10.3389/fphys.2018.00210 PMC5854678

[26] Tan Z, Cao L, Wu Y, Wang B, Song Z, Yang J, et al. Bisecting GlcNAc modification diminishes the pro-metastatic functions of small extracellular vesicles from breast cancer cells. J Extracell Vesicles. 2020;10(1):e12005. 33304474 10.1002/jev2.12005 PMC7710122

[27] Yoshihara K, Shahmoradgoli M, Martínez E, Vegesna R, Kim H, Torres-Garcia W, et al. Inferring tumour purity and stromal and immune cell admixture from expression data. Nat Commun. 2013;4:2612. 24113773 10.1038/ncomms3612 PMC3826632

[28] Schmittgen TD, Livak KJ. Analyzing real-time PCR data by the comparative C(T) method. Nat Protoc. 2008;3(6):1101–8. 18546601 10.1038/nprot.2008.73

[29] Zhou X, Zhang J, Song Z, Lu S, Yu Y, Tian J, et al. ExoTracker: a low-pH-activatable fluorescent probe for labeling exosomes and monitoring endocytosis and trafficking. Chem Commun (Camb). 2020;56(94):14869–72. 33174884 10.1039/d0cc06208a

[30] Chatila T, Silverman L, Miller R, Geha R. Mechanisms of T cell activation by the calcium ionophore ionomycin. J Immunol. 1989;143(4):1283–9. 2545785

[31] Fang Z, Qin H, Mao J, Wang Z, Zhang N, Wang Y, et al. Glyco-Decipher enables glycan database-independent peptide matching and in-depth characterization of site-specific N-glycosylation. Nat Commun. 2022;13(1):1900. 35393418 10.1038/s41467-022-29530-y PMC8990002

[32] Illiano M, Sapio L, Salzillo A, Capasso L, Caiafa I, Chiosi E, et al. Forskolin improves sensitivity to doxorubicin of triple negative breast cancer cells via protein kinase A-mediated ERK1/2 Inhibition. Biochem Pharmacol. 2018;152:104–13. 29574069 10.1016/j.bcp.2018.03.023

[33] Huang Y, Zhang HL, Li ZL. FUT8-mediated aberrant N-glycosylation of B7H3 suppresses the immune response in triple-negative breast cancer. Nat Commun. 2021;12(1):2672. 33976130 10.1038/s41467-021-22618-x PMC8113546

[34] Shi C, Wang Y, Wu M, Chen Y, Liu F, Shen Z, et al. Promoting anti-tumor immunity by targeting TMUB1 to modulate PD-L1 polyubiquitination and glycosylation. Nat Commun. 2022;13(1):6951. 36376293 10.1038/s41467-022-34346-x PMC9663433

[35] Xu S, Chen X, Fang J, Chu H, Fang S, Zeng L, et al. Comprehensive analysis of 33 human cancers reveals clinical implications and immunotherapeutic value of the solute carrier family 35 member A2. Front Immunol. 2023;14:1155182. 37275857 10.3389/fimmu.2023.1155182 PMC10232969

[36] Dong H, Strome SE, Salomao DR, Tamura H, Hirano F, Flies DB, et al. Tumor-associated B7-H1 promotes T-cell apoptosis: a potential mechanism of immune evasion. Nature medicine. Nat Med. 2002;8(8):793–800. 12091876 10.1038/nm730

[37] Cheng XT, Xie YX. Characterization of LAMP1-labeled nondegradative lysosomal and endocytic compartments in neurons. J Cell Biol. 2018;217(9):3127–39. 29695488 10.1083/jcb.201711083 PMC6123004

[38] Yin Z, Yu M, Ma T, Zhang C, Huang S, Karimzadeh MR, et al. Mechanisms underlying low-clinical responses to PD-1/PD-L1 blocking antibodies in immunotherapy of cancer: a key role of Exosomal PD-L1 [J]. J Immunother Cancer. 2021;9(1):e001698. 33472857 10.1136/jitc-2020-001698 PMC7818841

[39] Théry C, Witwer KW. Minimal information for studies of extracellular vesicles 2018 (MISEV2018): a position statement of the international society for extracellular vesicles and update of the MISEV2014 guidelines. J Extracell Vesicles. 2018;7(1):1535750. 30637094 10.1080/20013078.2018.1535750 PMC6322352

[40] Zhang X, Zhang C, Qiao M, Cheng C, Tang N, Lu S, et al. Depletion of BATF in CAR-T cells enhances antitumor activity by inducing resistance against exhaustion and formation of central memory cells. Cancer Cell. 2022;40(11):1407–e227. 36240777 10.1016/j.ccell.2022.09.013

[41] Sun K, Zhi Y, Ren W, Li S, Zheng J, Gao L, et al. Crosstalk between O-GlcNAcylation and ubiquitination: a novel strategy for overcoming cancer therapeutic resistance. Exp Hematol Oncol. 2024;13(1):107. 39487556 10.1186/s40164-024-00569-5 PMC11529444

[42] Büll C, Boltje TJ, Balneger N, Weischer SM, Wassink M, van Gemst JJ, et al. Sialic acid Blockade suppresses tumor growth by enhancing T-cell-Mediated tumor immunity. Cancer Res. 2018;78(13):3574–88. 29703719 10.1158/0008-5472.CAN-17-3376

[43] Xiao H, Woods EC, Vukojicic P, Bertozzi CR. Precision glycocalyx editing as a strategy for cancer immunotherapy. Proc Natl Acad Sci U S A. 2016;113(37):10304–9. 27551071 10.1073/pnas.1608069113 PMC5027407

[44] Gray MA, Stanczak MA, Mantuano NR, Xiao H, Pijnenborg JFA, Malaker SA, et al. Targeted glycan degradation potentiates the anticancer immune response in vivo. Nat Chem Biol. 2020;16(12):1376–84. 32807964 10.1038/s41589-020-0622-x PMC7727925

[45] Pei J, Wang G, Feng L, Zhang J, Jiang T, Sun Q, et al. Targeting lysosomal degradation pathways: new strategies and techniques for drug discovery. J Med Chem. 2021;64(7):3493–507. 33764774 10.1021/acs.jmedchem.0c01689

[46] Yoshimura M, Nishikawa A, Ihara Y, Taniguchi S, Taniguchi N. Suppression of lung metastasis of B16 mouse melanoma by N-acetylglucosaminyltransferase III gene transfection. Proc Natl Acad Sci U S A. 1995;92(19):8754–8. 7568011 10.1073/pnas.92.19.8754 PMC41045

[47] Schachter H. Biosynthetic controls that determine the branching and microheterogeneity of protein-bound oligosaccharides. Biochem Cell Biol. 1986;64(3):163–81. 3521675 10.1139/o86-026

[48] Tan Z, Ning L, Cao L, Zhou Y, Li J, Yang Y, et al. Bisecting GlcNAc modification reverses the chemoresistance via attenuating the function of P-gp. Theranostics. 2024;14(13):5184–99. 39267774 10.7150/thno.93879 PMC11388069

[49] Li Z, Zhang N, Dong Z, Wang X, Zhou J, Gao J, et al. Integrating transcriptomics, glycomics and glycoproteomics to characterize hepatitis B virus-associated hepatocellular carcinoma. Cell Commun Signal. 2024;22(1):200. 38561745 10.1186/s12964-024-01569-y PMC10983713

[50] Kariya Y, Gu J, Kariya Y. Integrin α6β4 confers doxorubicin resistance in cancer cells by suppressing Caspase-3-Mediated apoptosis: involvement of N-Glycans on β4 integrin subunit. Biomolecules. 2023;13(12). 10.3390/biom13121752 PMC10741852 38136623

[51] Sun C, Mezzadra R, Schumacher TN. Regulation and function of the PD-L1 checkpoint. Immunity. 2018;48(3):434–52. 29562194 10.1016/j.immuni.2018.03.014 PMC7116507

[52] Cui Y, Li J, Zhang P, Yin D, Wang Z, Dai J, et al. B4GALT1 promotes immune escape by regulating the expression of PD-L1 at multiple levels in lung adenocarcinoma. J Exp Clin Cancer Res. 2023;42(1):146. 37303063 10.1186/s13046-023-02711-3 PMC10259029

[53] Seo N, Akiyoshi K, Shiku H. Exosome-mediated regulation of tumor immunology. Cancer Sci. 2018;109(10):2998–3004. 29999574 10.1111/cas.13735 PMC6172045

[54] Theodoraki MN, Yerneni SS, Hoffmann TK, Gooding WE, Whiteside TL. Clinical significance of PD-L1(+) exosomes in plasma of head and neck cancer patients. Clin Cancer Res. 2018;24(4):896–905. 29233903 10.1158/1078-0432.CCR-17-2664 PMC6126905

[55] Chen G, Huang AC, Zhang W, Zhang G, Wu M, Xu W, et al. Exosomal PD-L1 contributes to immunosuppression and is associated with anti-PD-1 response. Nature. 2018;560(7718):382–6. 30089911 10.1038/s41586-018-0392-8 PMC6095740

[56] 53, Ricklefs FL, Alayo Q, Krenzlin H, Mahmoud AB, Speranza MC, Nakashima H, et al. Immune evasion mediated by PD-L1 on glioblastoma-derived extracellular vesicles. Sci Adv. 2018;4(3):eaar2766. 29532035 10.1126/sciadv.aar2766 PMC5842038

[57] Stanczak MA, Rodrigues Mantuano N, Kirchhammer N, Sanin DE, Jacob F, Coelho R, et al. Targeting cancer glycosylation repolarizes tumor-associated macrophages allowing effective immune checkpoint Blockade. Sci Transl Med. 2022;14(669):eabj127. 10.1126/scitranslmed.abj1270 PMC9812757 36322632

[58] Krug J, Rodrian G, Petter K, Yang H, Khoziainova S, Guo W, et al. N-glycosylation regulates intrinsic IFN-γ resistance in colorectal cancer: implications for immunotherapy. Gastroenterology. 2023;164(3):392–e4065. 36402190 10.1053/j.gastro.2022.11.018 PMC10009756

[59] Yin Y, Feng W, Chen J, Chen X, Wang G, Wang S, et al. Immunosuppressive tumor microenvironment in the progression, metastasis, and therapy of hepatocellular carcinoma: from bench to bedside. Exp Hematol Oncol. 2024;13(1):72. 39085965 10.1186/s40164-024-00539-x PMC11292955

