# Supplementary material for: Bisecting GlcNAc enhances CD8+ T cell-mediated killing of breast cancer by suppressing PD-L1 expression and its binding to PD-1
Source: Exp Hematol Oncol. 2025 Aug 4;14:102. doi: 10.1186/s40164-025-00693-w (PMC12323189; doi:10.1186/s40164-025-00693-w)
Supplement: Supplementary file 1 — Supplementary Material 1 [file 40164_2025_693_MOESM1_ESM.docx]

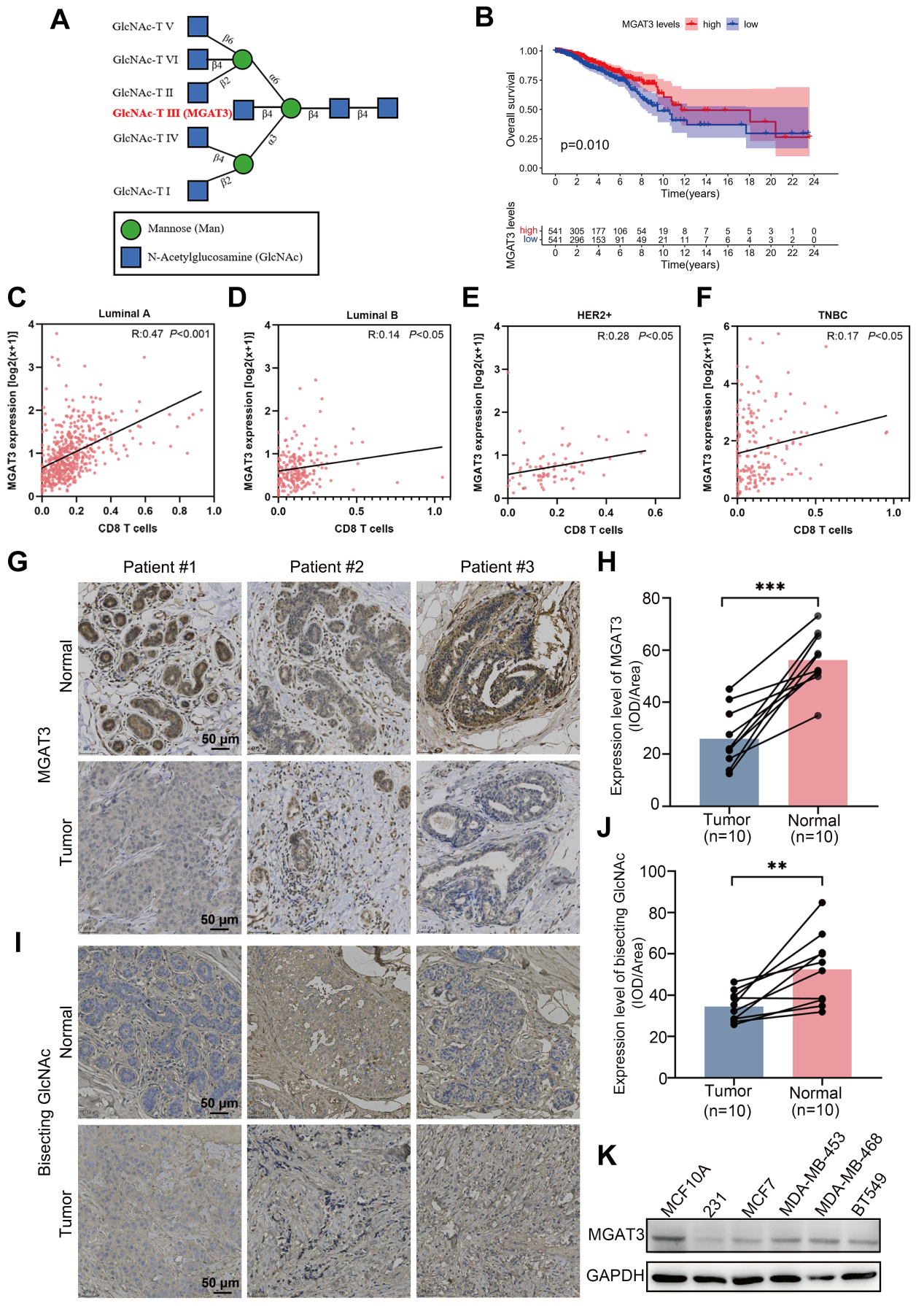


**Fig. S1 Levels of MGAT3 and bisecting GlcNAc in BC tissues and cell lines. A.** The schematic diagram of the bisecting GlcNAc structure catalyzed by MGAT3. **B.** The overall survival curves of BC samples from TCGA database was stratified by the median expression of MGAT3. The relationship between MGAT3 expression and CD8 T cell infiltration in **(C)** luminal A, **(D)** luminal B, **(E)** HER2+, and **(F)** TNBC was analyzed by stratification. Representative IHC images and comparison of expression levels of **(G&H)** MGAT3 and **(I&J)** bisecting GlcNAc in normal and tumor tissues of BC patients. **K.** Expression of MGAT3 in normal breast epithelial and BC cell lines.


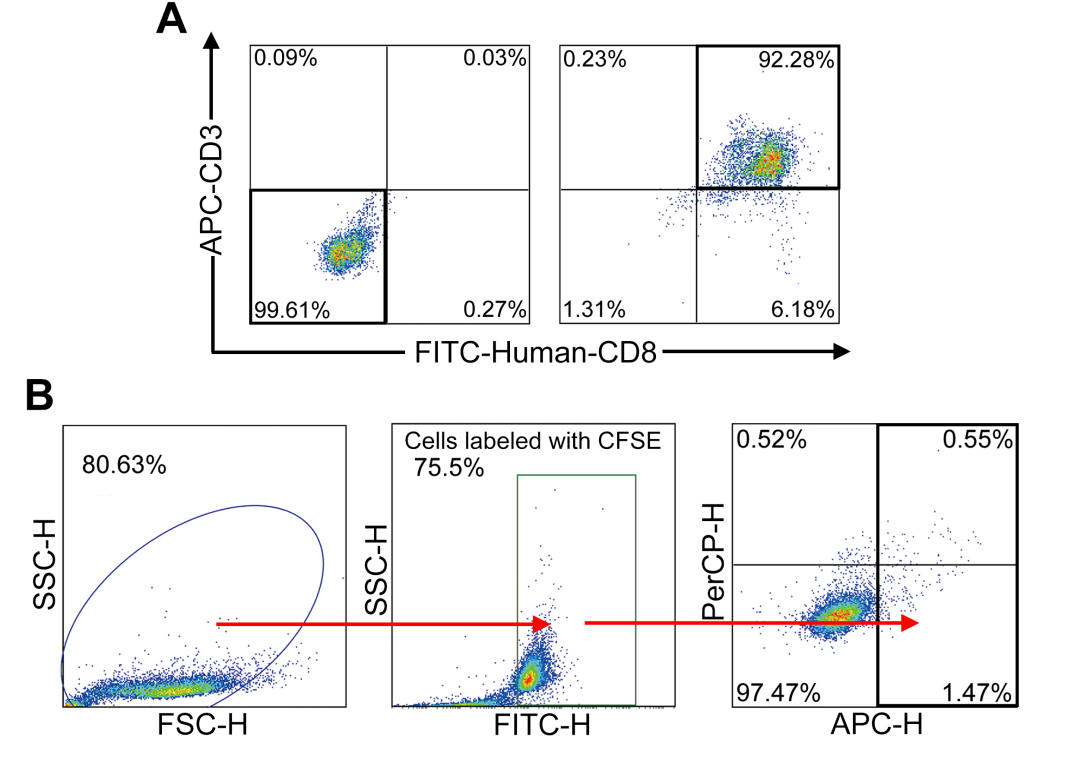


**Fig. S2 Flow cytometry gating strategy.** **A.** CD8^+^ T cells isolated from human PBMCs were identified by flow cytometry (left panel：CD8^+^ T magnetic bead sorting negative cells; right panel: CD8^+^ T magnetic bead sorting positive cells). **B.** Schematic diagram of the gating strategy for detecting apoptosis in CD8^+^ T cell-mediated killing (Step 1: Gating the main cell population; Step 2: gating CFSE-labeled tumor cells; Step 3: gating the early apoptotic population as Annexin V⁺/7-AAD⁻, gating the terminal apoptotic population as Annexin V⁺/7-AAD^+^).


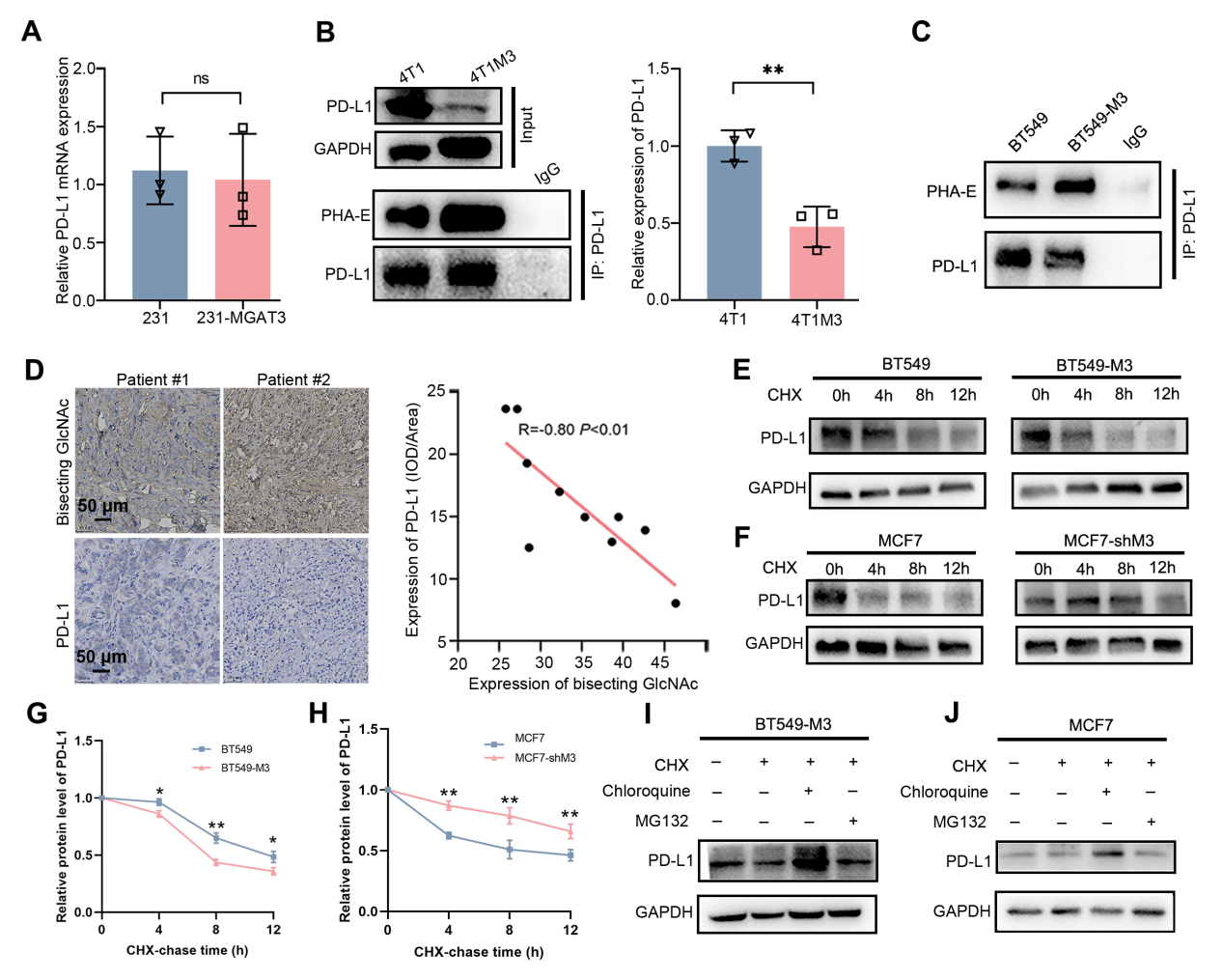


**Fig. S3 Bisecting GlcNAc promotes lysosomal degradation of PD-L1. A.** Expression of PD-L1 mRNA in 231 and 231-MGAT3 was analysed by qRT‒PCR. Expression of PD-L1 protein in **(B)** 4T1 /4T1M3 and **(C)** BT549/BT549-M3 were analysed by western blot. **D.** The representative IHC images of BC tissue samples (left panel) and correlation between bisecting GlcNAc and PD-L1 expression (right panel). CHX (20 μM) chase analysis of PD-L1 expression in **(E&G)** BT549/ BT549-M3 and **(F&H)** MCF7/ MCF7-shM3 cells. PD-L1 expression in **(I)** BT549-M3 and **(J)** MCF7 cells treated with chloroquine or MG132.

**
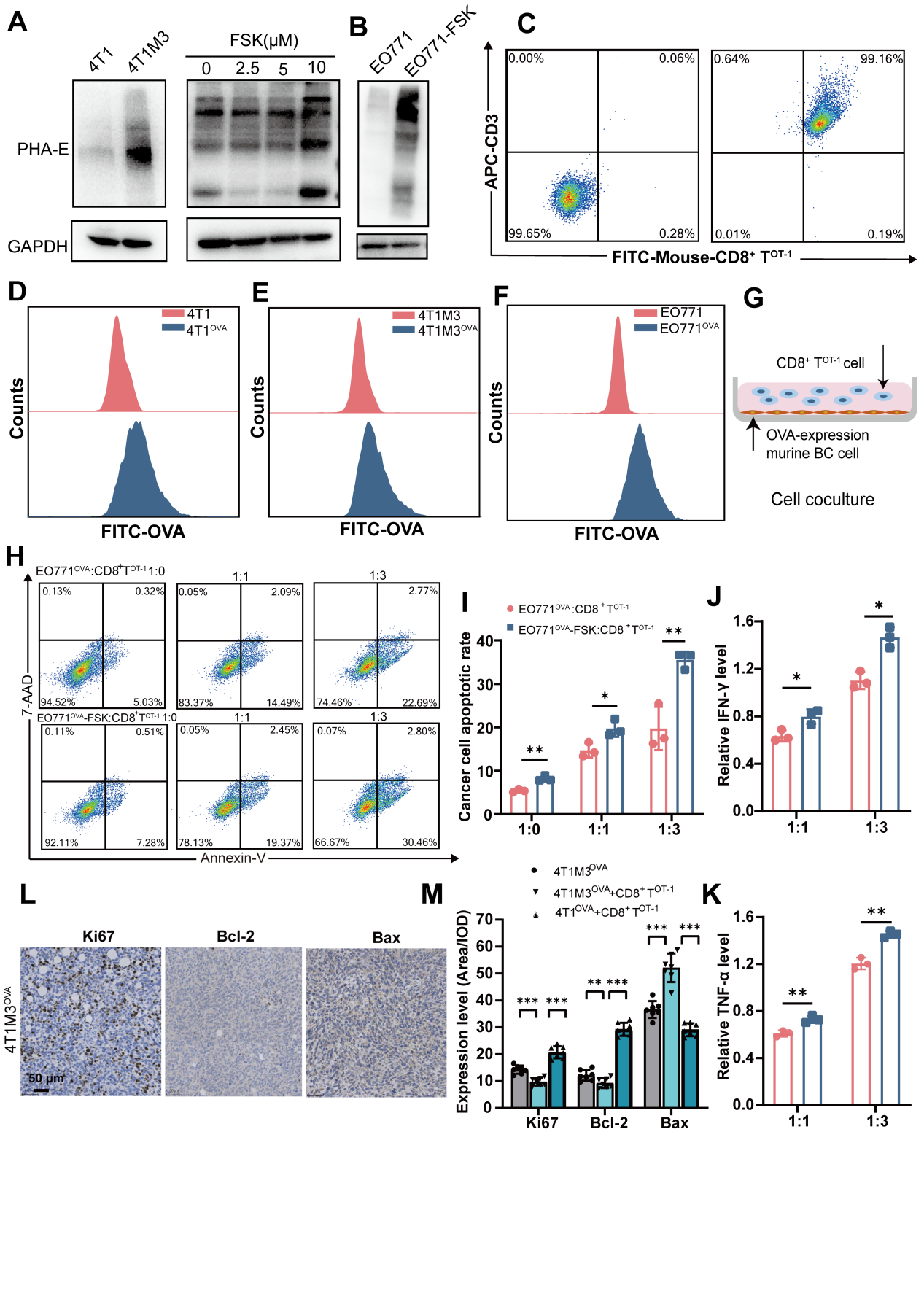
**

**Fig. S4 CD8^+^ T^OT-1^ cell-mediated killing of BC cells. A.** The level of bisecting GlcNAc in 4T1, MGAT3-overexpressing (4T1M3), and FSK (FSK)-treated 4T1 (4T1-FSK) cells. **B.** The level of bisecting GlcNAc in EO771 and FSK-treated EO771 cells. **C.** CD8^+^ T^OT-1^ cells isolated from OT-1 mice were identified by flow cytometry. OVA overexpressed **(D)** 4T1, **(E)** 4T1M3, and **(F)** EO771 were detected by flow cytometry. **G.** The diagram of CD8^+^ T^OT-1^ cell co-culture with murine BC cells *in vitro*. **H&I.** Flow cytometry was used to detect the apoptosis rates of EO771^OVA^ and EO771^OVA^-FSK cells. **J&K.** Levels of IFN-γ and TNF-α in co-culture medium were measured by ELISA. **L.** Representative IHC images in 4T1M3^OVA^ group. **M.** Quantitative analysis of Ki67, Bcl-2, and Bax levels.


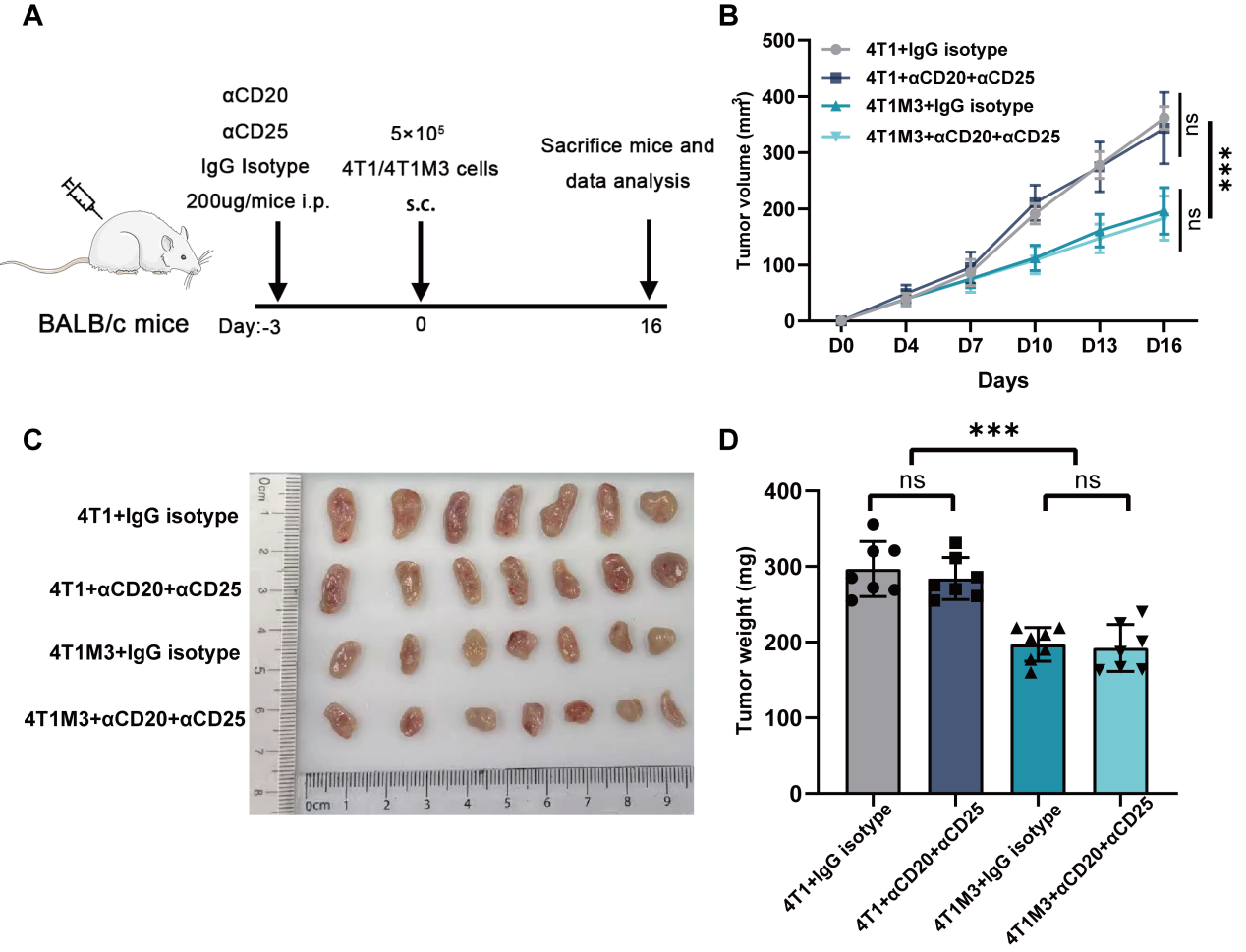
、**Fig. S5 The effect of B/Treg cells depletion on the antitumor efficacy of MGAT3. A.** The schematic illustration of experimental design. **(B)** Tumor growth curves, **(C)** tumor images, and **(D)** tumor weight.


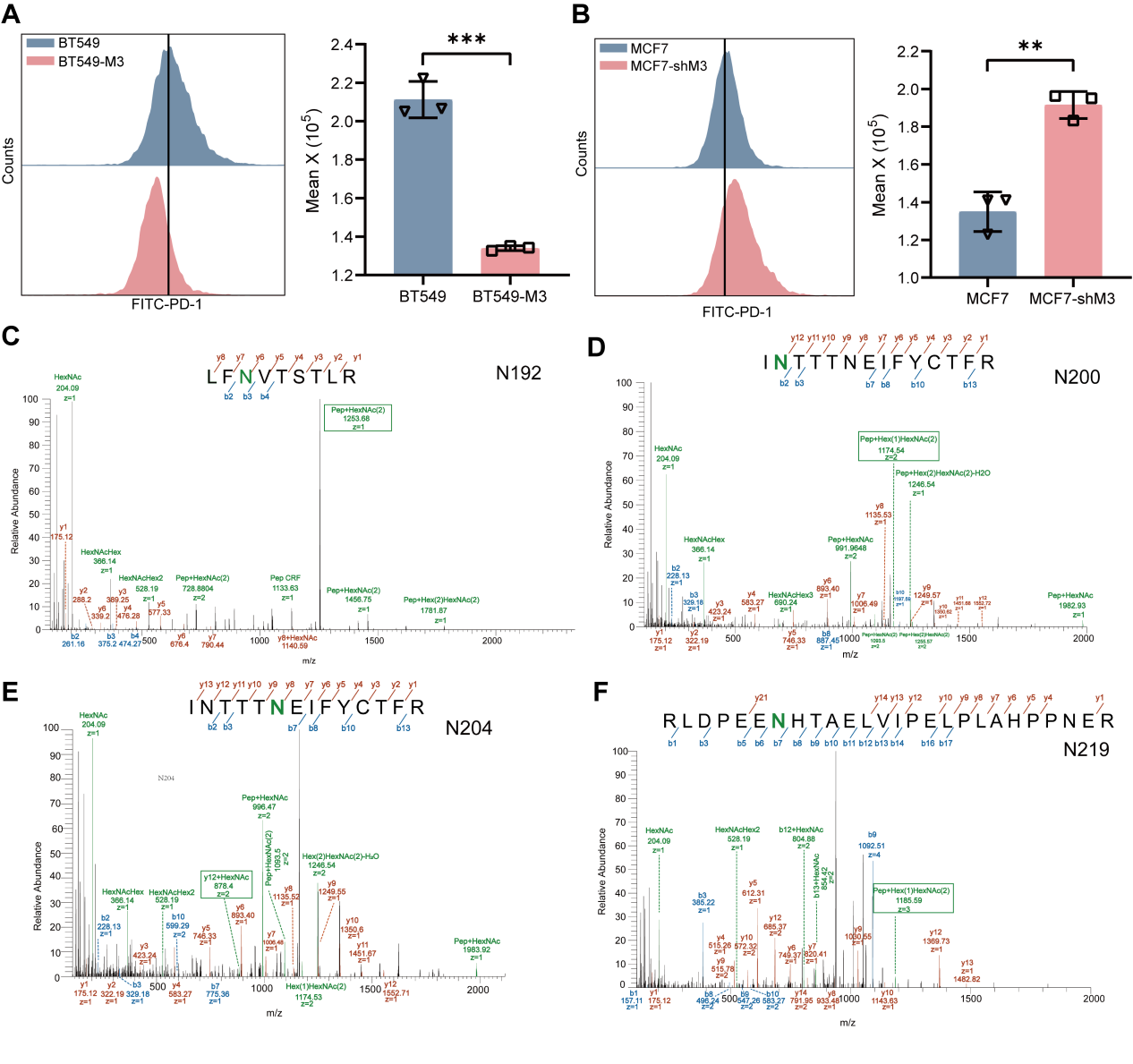


**Figure S6. The bisecting GlcNAc modification affects PD-L1 binding to PD-1.** The recombinant PD-1 binding to PD-L1 on the membrane of **(A)** BT549/ BT549-M3 or **(B)** MCF7/ MCF7-shM3 cells was detected by flow cytometry. **C-F.** Identification of N-glycosylation sites on PD-L1.
